# Digital pathology with artificial intelligence analysis provides insight to the efficacy of anti-fibrotic compounds in human 3D MASH model

**DOI:** 10.1038/s41598-024-55438-2

**Published:** 2024-03-11

**Authors:** Radina Kostadinova, Simon Ströbel, Li Chen, Katia Fiaschetti‑Egli, Jana Gadient, Agnieszka Pawlowska, Louis Petitjean, Manuela Bieri, Eva Thoma, Mathieu Petitjean

**Affiliations:** 1 PharmaNest, Princeton, NJ USA; 2 InSphero AG, Wagistrasse 27A, Schlieren, Switzerland

**Keywords:** Metabolic syndrome, Endocrine system and metabolic diseases, Diseases, Drug discovery, Drug screening, Target validation

## Abstract

Metabolic dysfunction-associated steatohepatitis (MASH) is a severe liver disease characterized by lipid accumulation, inflammation and fibrosis. The development of MASH therapies has been hindered by the lack of human translational models and limitations of analysis techniques for fibrosis. The MASH three-dimensional (3D) InSight™ human liver microtissue (hLiMT) model recapitulates pathophysiological features of the disease. We established an algorithm for automated phenotypic quantification of fibrosis of Sirius Red stained histology sections of MASH hLiMTs model using a digital pathology quantitative single-fiber artificial intelligence (AI) FibroNest™ image analysis platform. The FibroNest™ algorithm for MASH hLiMTs was validated using anti-fibrotic reference compounds with different therapeutic modalities-ALK5i and anti-TGF-β antibody. The phenotypic quantification of fibrosis demonstrated that both reference compounds decreased the deposition of fibrillated collagens in alignment with effects on the secretion of pro-collagen type I/III, tissue inhibitor of metalloproteinase-1 and matrix metalloproteinase-3 and pro-fibrotic gene expression. In contrast, clinical compounds, Firsocostat and Selonsertib, alone and in combination showed strong anti-fibrotic effects on the deposition of collagen fibers, however less pronounced on the secretion of pro-fibrotic biomarkers. In summary, the phenotypic quantification of fibrosis of MASH hLiMTs combined with secretion of pro-fibrotic biomarkers and transcriptomics represents a promising drug discovery tool for assessing anti-fibrotic compounds.

## Introduction

Metabolic dysfunction-associated steatohepatitis (MASH) is currently the most common liver disorder in Western Countries and its societal health effects are rising with its prevalence. MASH is the progressive form of metabolic dysfunction-associated steatotic liver disease (MASLD), which is characterized by hepatic steatosis, hepatic injury and inflammation. As hepatic injury progresses, MASH can develop into advanced fibrosis and cirrhosis, where hepatic vasculature and architecture is disrupted, which can eventually lead to liver failure and death. Fibrosis has been shown to be the strongest predictor of mortality, establishing it as a critical therapeutic target in MASH. Currently, there are no approved drugs for MASH, thus highlighting an urgent unmet need. Although our clinical understanding of MASH has advanced significantly in recent years, all therapies which have been tested in clinical trials for the treatment of liver fibrosis have so far not gained Food and Drug Administration (FDA) regulatory approval^1^. Part of this failure stems from the lack of human-relevant translational preclinical models which are predictive of clinical outcomes. Another reason is that the initiation and progression of MASH often takes decades and involves a complex interplay between different organs^2^ and cell types^3^. Current models are unable to mimic all these stages of the disease, which is particularly challenging due to the diverse nature of the mechanisms involved, including environmental and genetic factors^4,5^. Animal models are still used in preclinical studies^6,7^; however, they do not represent human-specific effects due to differences in metabolism, nutrition and fibrosis^8,9^. They also need strong stimuli such as hepatotoxicants to induce liver damage^10^. Efficacy testing of MASH therapeutics is performed in very young animals which do not necessarily reflect the clinical MASH pathology^11^. The European Medicines Agency (EMA) supports the 3Rs principles-replace, reduce and refine and the implementation of new approaches such as in vitro human cell-based models to minimize animal testing during drug development. Furthermore the FDA Modernization Act 2.0 considers translational and cutting-edge testing methods such as human cells, organoids and organs-on-chips, and advanced artificial intelligence (AI) methods for preclinical drug safety and efficacy assessment of new medicines^12^. Therefore, the success of new therapies relies on the availability of relevant and predictive human translational in vitro MASH models that accurately mimic the underlying mechanisms of the disease.

One promising MASH model is the three-dimensional (3D) InSight™ human liver microtissues (hLiMTs) which are comprised of primary human hepatocytes (PHH) and non-parenchymal cells such as hepatic stellate cells (HSC), Kupffer cells (KC) and liver sinusoidal endothelial cells (LEC). hLiMTs have been shown to be translational preclinical models for advanced MASH when exposed to free fatty acids (FFAs), sugars and lipopolysaccharide (LPS)^13,14^. High levels of FFAs cause lipid accumulation followed by damage to the hepatocytes by lipo-apoptosis^15^, which then results in the activation of KC, triggering a pro-inflammatory response and activation of HSC. Activated HSC differentiate into a fibrogenic myofibroblast like phenotype that produces smooth muscle actin and collagens, particularly type I, III and VI, causing excessive deposition of extracellular matrix, which is conducive for the development of fibrosis during both experimental and chronic human liver injury^16,17^. The 3D hLiMTs can be maintained in culture for over four weeks and are well characterized with respect to adenosine triphosphate (ATP) levels, albumin secretion, native human liver gene expression, histological assessment and with the expression of the bile salt export pump (BSEP) and the multidrug resistance protein 2 (MRP2)^18,19^.

**Figure 1:**
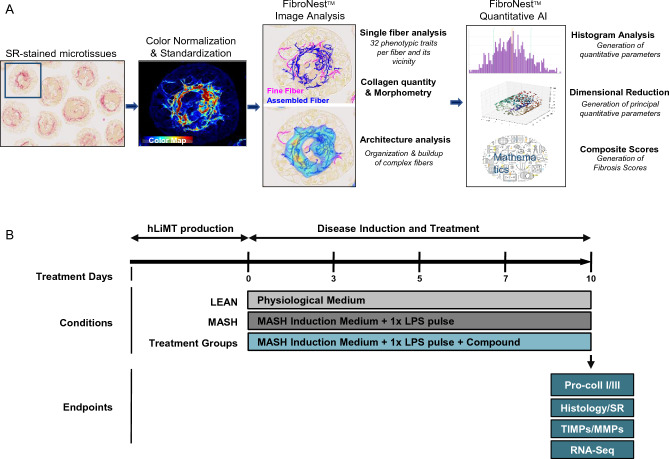
Overview of FibroNest™ platform workflow for fibrosis quantification and MASH induction method. (**A**) FibroNest™ quantitative image analysis workflow and method. (**B**) Scheme for MASH induction in hLiMTs. Disease induction and treatment start at day 0 after the production of the hLiMTs. The three treatment conditions are: LEAN, MASH and MASH in the presence of test compounds. After each medium exchange on days 0, 3, 5 and 7, the compounds and vehicle controls (DMSO or PBS) were applied. FFAs supplementation occurred on each treatment day, while stimulation with LPS took place only once during the 10-day treatment period. Cell culture supernatants and/or microtissues were collected on days 5, 7 and 10. All the end points related to pro-collagen type I/III (pro-coll I/III), Histology/SR, TIMPs/matrix metalloproteinases (MMPs) and RNA sequencing (RNA-Seq) were performed on day 10. All conditions were normalized to 0.2% DMSO or PBS.

To evaluate the effect of a preclinical/clinical candidate in a model, whether it is in vivo or in vitro, the analysis technique used is key in studying the disease development or regression. Although noninvasive tests (NITs) such as transient elastography (FibroScan®), magnetic resonance elastography, Fibrosis-4 (FIB-4) index and secretion of pro-fibrotic biomarkers such as tissue inhibitor of metalloproteinase (TIMP)-1, amino-terminal pro-peptide of type III pro-collagen (PIIINP) and hyaluronic acid (HA) present in the clinical enhanced liver fibrosis (ELF™) test, are preferred, histological assessment of Sirius Red (SR) stained slices of human liver biopsies via semiquantitative scoring systems remain the current gold standard for clinical diagnosis of MASH. Human-based scoring mechanisms have been shown to be unreliable with both high intra- and inter-operator variabilities. On the other hand, traditional computation fibrosis analysis, such as overall collagen area ratio, is blunt and insensitive to nuanced mild drug effects in early clinical treatments. Therefore, there is a need for highly sensitive quantitative fibrosis analysis which allows the identification of not only the total level of fibrosis but also the phenotypes of fibrosis. Although not yet used in routine clinical practice, advances in AI image analysis show great promise in characterizing architectural features of fibrosis at the individual collagen fiber level. Quantification and calculation of different variables in collagen content, morphology and architecture can be used to establish algorithm-based quantitative fibrosis scores, which have been validated against fibrosis stage in MASLD. AI is being explored to further refine and develop quantitative fibrosis scoring methods. One of these new technologies is FibroNest™^20–25^. This is a cloud-based, digital pathology (DP) platform which quantifies the histological phenotypes of MASH fibrosis (e.g., collagen fiber width) to calculate continuous severity scores for collagen content (12 histological traits), collagen fiber morphometry (13 traits) and fibrosis architecture (7 traits, Fig. 1A). These 32 phenotypic traits are combined into normalized phenotypic fibrosis composite score (Ph-FCS). This approach allows simultaneous assessment of architectural and morphological changes as well as collagen content, allowing for a more nuanced analysis of fibrosis. In turn, this has the potential for increased sensitivity to detect and understand anti-fibrotic drug effects. FibroNest™ platform has been validated in recent years based on the quantification of fibrosis of many biopsies from healthy and MASLD patients, as well as patients undergoing anti-MASH compound treatment^20–24^. For better translation of the compound efficacy findings the same or similar DP/AI platforms should be used for phenotypic quantification of fibrosis in both preclinical and clinical tests.

In this study, we have combined the use of the MASH hLiMT model and the FibroNest™ DP/AI platform to analyze the anti-fibrotic drug effects of two reference control anti-fibrotic compounds, activin receptor-like kinase 5 inhibitor (ALK5i)^26^ and anti-TGF-β antibody (Ab)^27^, and the clinically tested drugs, Firsocostat (an acetyl-CoA carboxylase inhibitor^28^) and Selonsertib (an apoptosis signal-regulating kinase 1 inhibitor^1,29–31^). The main aims were to assess the ability of the FibroNest™ platform to quantify the severity of fibrosis and drug effects in MASH hLiMTs based on SR stained histology slides and to relate the efficacy of these drug candidates in the preclinical model to their impact on fibrosis in clinical trials. In addition, we wanted to demonstrate that depending on the mechanism of action of the anti-fibrotic compounds the assessment of their effect using only the secretion of pro-fibrotic markers or transcriptomics might not be sufficiently informative. Therefore, additionally the quantification of deposited fibrillated collagens using AI FibroNest™ image analysis platform should be performed to correctly assess the efficacy of the anti-fibrotic compounds, which mechanism of action is to affect the formation, degradation and remodeling, of the extracellular matrix.

## Results

### Mechanistic investigation of the effect of anti-fibrotic reference compounds ALK5i and anti-TGF-β Ab

#### Alignment of the secretion of pro-fibrotic markers and transcriptomics data with phenotypic quantification of fibrosis of ALK5i

**Figure 2:**
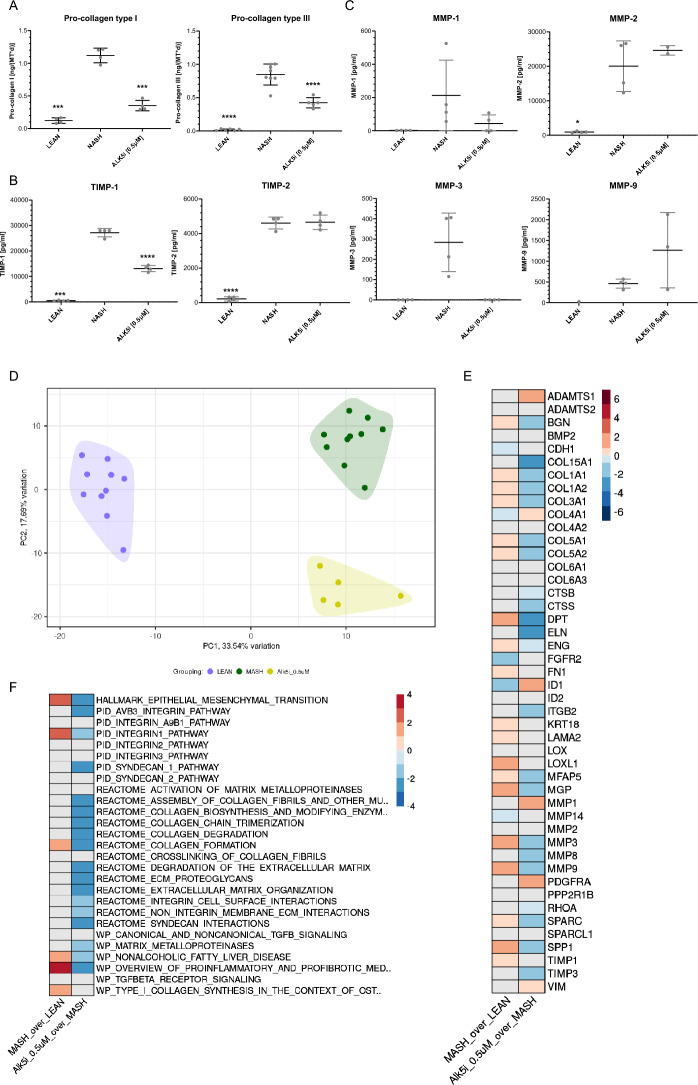
ALK5i treatment leads to decreased fibrosis in MASH hLiMTs. (**A**) Pro-collagen type I and pro-collagen type III, (**B**) TIMP-1 and TIMP-2 and (**C**) MMP-1, MMP-2, MMP-3 and MMP-9 in LEAN, MASH and MASH treated with 0.5 µM ALK5i hLiMTs on day 10. Mean ± SD, n = 4 microtissues/group, except for pro-collagen type III, where n = 6 microtissues/group were used. The data points below the lowest standard value were excluded from the data set. *p ≤ 0.05 (one-way ANOVA (analysis of variance), Welch's correction) MASH vs MASH + ALK5i. (**D**) Principal component analysis (PCA) plot presenting intervention wise grouping of LEAN, MASH and MASH treated with 0.5 µM ALK5i hLiMTs on day 10. (**E**) Summary heatmap presenting differential expression analysis (DEA) results on day 10 for the selected genes related to fibrosis (Supplementary table 4) in MASH compared to LEAN controls (left column) and in MASH treated with 0.5 µM ALK5i compared to MASH counterparts (right column) hLiMTs. Color scale represents Log2FC values derived from DEA (Wald test, Benjamini–Hochberg correction). Log2FC values associated with false discovery rate (FDR) > 0.01 were overwritten with zero and are depicted in grey. Color scale range was determined based on the spread of the log2FC values for all genes investigated in the study. (**F**) Summary heatmap presenting gene set enrichment analysis (GSEA) results on day 10 for the selected gene sets related to fibrosis (Supplementary table 5) in MASH compared to LEAN controls (left column) and in MASH treated with 0.5 µM ALK5i compared to MASH counterparts (right column) hLiMTs. Color scale represents normalized enrichment score (NES) values derived from GSEA (Kolmogorov–Smirnov-like statistic + permutation test, Benjamini–Hochberg correction). NES values associated with FDR > 0.05 were overwritten with zero and are depicted in grey. Color scale range was determined based on the spread of the NES values for all gene sets investigated in the study.

The induction of MASH in hLiMTs medium containing high levels of sugars, insulin, fatty acids and LPS pulse (Fig. 1B) resulted in an ~ 8–10-fold higher synthesis and secretion of pro-collagen type I and III than LEAN hLiMTs. ALK5i treatment decreased the synthesis and secretion of pro-collagen type I and III in MASH hLiMTs by ~ 70% and ~ 60%, respectively at day 10 (Fig. 2A). There was a significant increase in the secretion of TIMP-1 and TIMP-2 (Fig. 2B), as well as matrix metalloproteinase (MMP)-1, -2, -3 and -9 (Fig. 2C) in MASH hLiMTs compared to LEAN hLiMTs. ALK5i treatment decreased the secretion of TIMP-1, MMP-1 and MMP-3 but not TIMP-2 and MMP-2 on day 10 in MASH hLiMTs (Fig. 2B and 2C). The ALK5i treatment resulted in a trend towards increasing MMP-9 secretion; however, this observed effect did not reach statistical significance (Fig. 2C).

To understand further the anti-fibrotic mechanisms of action of ALK5i in MASH hLiMTs we performed whole transcriptomic profiling of individual microtissues using templated oligo-sequencing (TempO-Seq®). PC1 and PC2 plots revealed clustering of LEAN, MASH and MASH treated with ALK5i groups (Fig. 2D). We showed an alignment of the protein secretion of pro-fibrotic markers with their gene expression levels. There was a significant increase of the expression of key pro-fibrotic genes of fibrillar collagens such as COL1A1, COL1A2, COL3A1, COL5A1 and COL5A2 in MASH hLiMTs, which decreased in MASH treated with ALK5i group similar to its effects on pro-collagen type I and III secretion (Fig. 2A and E). The fibrillar collagens genes such as COL4A1 and COL4A2, which are part of basement membrane, were down-regulated in MASH vs LEAN hLiMTs. Fibronectin 1 (FN1) and laminin subunit alpha 2 (LAMA2) genes, also part of basement membrane, were up-regulated in MASH vs LEAN hLiMTs conditions (Fig. 2E). Elastin (ELN) and FN1 genes, as part of ECM, were strongly down-regulated upon treatment with ALK5i. In addition, other pro-fibrotic genes such as secreted phosphoprotein 1 (SPP1, osteopontin 1), secreted protein, acidic and rich in cysteine (SPARC), dermatopontin (DPT), biglycan (BGN), microfibril associated protein 2 (MFAP5), matrix gla-protein (MGP) and endoglin (ENG) were significantly increased in MASH vs LEAN hLiMTs conditions and decreased by ALK5i treatment. Furthermore, in line with the protein secretion results, MMP-3 gene was down-regulated upon treatment with ALK5i compared to MASH condition (Fig. 2E). MMP-9, MMP-8 and TIMP-3 genes were significantly decreased by ALK5i as compared to MASH hLIMTs. Gene set enrichment analysis (GSEA) revealed that ALK5i treatment as compared to MASH condition decreased many pro-fibrotic pathways, including alpha v beta 3 (ABV3) integrin, integrin 1, syndecan 1, epithelial mesenchymal transition, degradation and organization of ECM, collagen fibers formation, degradation and assembly, collagen biosynthesis, collagen trimerization, crosslinking of collagen fibrils, MMPs, pro-inflammatory and pro-fibrotic pathways, ECM proteoglycans and wound healing (Fig. 2F). These results suggest that the anti-fibrotic mechanism of action of ALK5i relates to the decrease in collagen synthesis, assembly, remodeling and degradation.

**Figure 3:**
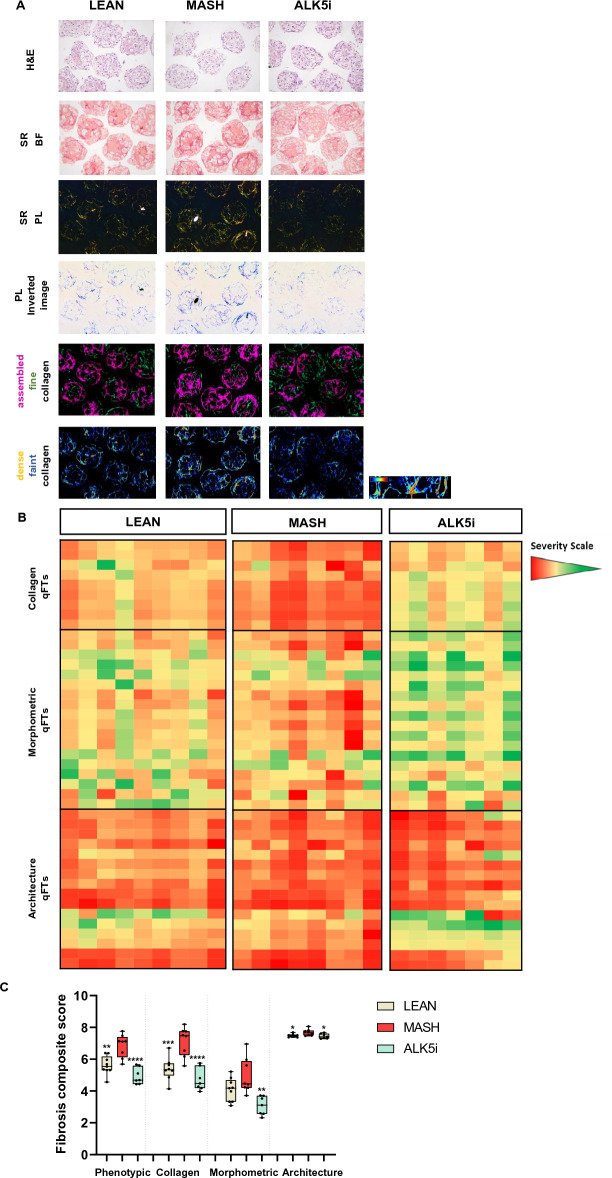
ALK5i treatment decreases the deposition of fibrillated collagens in MASH hLiMTs. (**A**) H&E and SR staining of deposited fibrillated collagens (SR-BF and SR-PL images) and their quantification by FibroNest™ II platform of LEAN, MASH and MASH treated with 0.5 µM ALK5i hLiMTs on day 10. Green is fine collagen; purple is assembled collagen and denotes the highly reticulated collagen; dark yellow and red indicate dense collagen and blue indicates faint collagen. The images were taken with 10 × magnification, scale bar: 100 µm. (**B**) Heatmap of the extent of fibrosis severity, ranging from green: faint collagen to red: dense collagen. Every column represents a hLiMT sample, and every row represents a different quantitative fibrosis traits (qFTs) that constitute the collagen, morphometric and architecture sub-phenotypes. (**C**) Ph-FCS, collagen-FCS, morphometric-FCS and architecture-FCS generated by 43 qFTs (Supplementary Table 1). Mean ± SD, n ≥ 7 microtissues/group, *p ≤ 0.05, **p ≤ 0.01, ***p ≤ 0.001, ****p ≤ 0.0001, (one-way ANOVA, Welch's correction), MASH vs LEAN or compound treatment.

To investigate whether the effect of ALK5i with respect to the production of collagens was in line with their deposition and degradation, histological slides of MASH hLiMTs sections were stained with SR to visualize collagen fiber deposition (Fig. 3A). The bright field (BF) and polarized light (PL) images of SR indicated an increased fibrosis in MASH conditions compared to LEAN and MASH treated with ALK5i hLiMTs. To comprehensively quantify tissue fibrosis, we leveraged a single-fiber DP/AI-based platform, FibroNest™, that extensively characterizes both absolute collagen content and important statistical features of the distributions of collagen fibers, morphometric and architectural phenotypes. There was basal amount of deposition of fine and faint collagen fibers (green and blue color fibers, respectively) in LEAN hLiMTs (Fig. 3A). In MASH hLiMTs, there was an increased collagen deposition and fibrillation compared to LEAN hLiMTs, whereby the amount of fine collagen (green color fibers) decreased, and the amount of assembled collagen (purple color fibers) increased (Fig. 3A). As expected, the severity of fibrosis decreased in MASH treated with ALK5i  hLiMTs compared to the MASH condition, as quantified using FibroNest™ severity scores. ALK5i treatment prevented the formation of assembled (complex, highly reticulated collagen), thick and dense collagens (purple, yellow and red color fibers, Fig. 3A), whereas the fine and faint collagens (green and blue color fibers) were detected at similar level in all groups. FibroNest™ version II software was used to quantify the extent and severity of fibrosis, which are presented as a heatmap (Fig. 3B and Supplementary Table 1). Each phenotypic quantitative fibrosis trait (qFT) is described individually for relative severity from least to most (green to red, respectively) for different fibrosis phenotypes, namely the collagen content, collagen fiber morphometry and fibrosis architecture in LEAN, MASH and MASH treated with ALK5i hLiMTs. For the generation of collagen-fibrosis composite score (FCS), morphometric-FCS or architecture-FCS, only the significantly changed 43 quantitative fibrosis traits (qFTs) in MASH hLiMTs vs LEAN or compound treatment were used (Fig. 3C). The anti-fibrotic effect of ALK5i was demonstrated by decrease of Ph-FCS, which is generated by the aggregate of the collagen, morphometric and architecture sub-phenotypes. Moreover, ALK5i treatment also significantly decreased each of these three sub-phenotypes. The most decreased qFTs below the LEAN hLiMTs group upon treatment with ALK5i were associated with collagen content (e.g. total collagen density area ratio, assembled collagen area ratio, and collagen structure index) and morphometry (e.g. total skeleton length, collagen width, collagen perimeter, area to perimeter ratio) (Supplementary Table 1). Under LEAN conditions, which represent the treatment with 0.2% DMSO vehicle control, a pronounced elevation in fibrosis architecture qFTs was evident. Therefore, minimal differences were observed between the MASH vs LEAN or MASH with ALK5i hLiMTs conditions with respect to architecture-FCS (Fig. 3C and Supplementary Table 1).

#### Alignment of the secretion of pro-fibrotic markers and transcriptomics data with phenotypic quantification of fibrosis upon treatment with anti-TGF-β Ab

**Figure 4:**
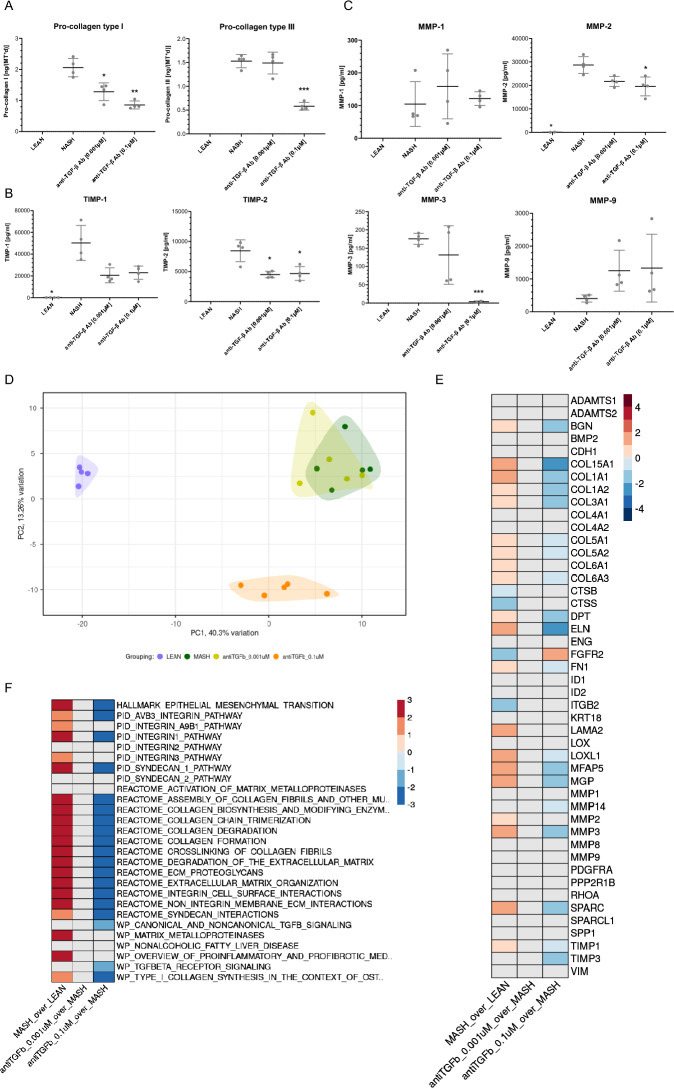
Anti-TGF-β Ab treatment leads to decreased fibrosis in MASH hLiMTs. (**A**) Pro-collagen type I and pro-collagen type III, (**B**) TIMP-1 and TIMP-2, (**C**) MMP-1, MMP-2, MMP-3 and MMP-9 in LEAN, MASH and MASH treated with 0.001 µM and 0.1 µM anti-TGF-β Ab hLiMTs on day 10. Mean ± SD, n = 4 microtissues/group, data points below the lowest standard value were excluded from the data set. *p ≤ 0.05 (one-way ANOVA, Welch's correction, MASH vs LEAN and compound treatment. (**D**) PCA plot presenting intervention wise-grouping of LEAN, MASH and MASH treated with anti-TGF-β Ab (0.001 µM and 0.1 µM) hLiMTs on day 10. (**E**) Summary heatmap presenting DEA results on day 10 for the selected genes related to fibrosis (Supplementary Table 4) in MASH hLiMTs compared to LEAN controls (column 1) and in MASH hLiMTs treated with anti-TGF-β Ab (0.001 µM and 0.1 µM) compared to MASH counterparts (column 2 and 3). Color scale represents Log2FC values derived from DEA (Wald test, Benjamini–Hochberg correction). Log2FC values associated with FDR > 0.01 were overwritten with zero and are depicted in grey. Color scale range was determined based on the spread of the log2FC values for all genes investigated in the study. (**F**) Summary heatmap presenting GSEA results on day 10 for the selected gene sets related to fibrosis (Supplementary table 5) in MASH hLiMTs compared to LEAN controls (column 1) and in MASH hLiMTs treated with anti-TGF-β Ab (0.001 µM and 0.1 µM) compared to MASH counterparts (column 2 and 3). Color scale represents NES values derived from GSEA (Kolmogorov–Smirnov-like statistic + permutation test, Benjamini–Hochberg correction). NES values associated with FDR > 0.05 were overwritten with zero and are depicted in grey. Color scale range was determined based on the spread of the NES values for all gene sets investigated in the study.

In MASH hLiMTs, there was a concentration-dependent decrease of the synthesis and secretion of pro-collagen type I by anti-TGF-β Ab at day 10 . Pro-collagen type III secretion was decreased on day 10 by 0.1 µM but not 0.001 µM anti-TGF-β Ab (Fig. 4A). Both concentrations of anti-TGF-β Ab significantly and markedly decreased TIMP-1 and TIMP-2 in MASH hLiMTs (Fig. 4B), which was not dose-dependent. While MMP-1 was not significantly altered by anti-TGF-β Ab, it did cause a dose-dependent decrease in the secretion of MMP-2- and MMP-3 (Fig. 4C). The treatment with anti-TGF-β Ab resulted in a trend to increase MMP-9 secretion compared to MASH hLiMT conditions; however, this effect did not reach statistical significance (Fig. 4C). The IgG control did not significantly affect the secretion of pro-collagen type I, III, TIMPs and MMPs (data not shown).

The results of the biochemical analyses were further confirmed by whole transcriptomic profiling of 0.001 µM and 0.1 µM anti-TGF-β Ab-treated MASH hLiMTs on day 10. PC1 and PC2 plots revealed clustering of LEAN and MASH with 0.1 µM anti-TGF-β Ab treatment groups (Fig. 4D). A concentration of 0.001 µM anti-TGF-β Ab did not cluster separately from the MASH group, probably due to a lack of potency on the gene expression at this low concentration. In MASH hLiMTs, there was an increase in the gene expression levels of fibrillar collagens such as COL1A1, COL1A2, COL3A1, COL5A1 and COL5A2, as well as non-fibrillar collagens, COL15A1 and COL6A3, which were significantly decreased upon 0.1 µM anti-TGF-β Ab treatment (Fig. 4E). Basement membrane genes such as LAMA2, FN1 and perlecan (HSPG2) were up-regulated in MASH vs LEAN hLiMT and the latter two genes down-regulated by 0.1 µM anti-TGF-β Ab (the data for HSPG2 are not shown). Furthermore MMP-3 and TIMP-1 gene expression levels increased in MASH hLiMTs were decreased upon treatment with 0.1 µM anti-TGF-β Ab similar to their protein secretion levels (Fig. 4C). In addition, other pro-fibrotic genes up-regulated in MASH hLiMTs as compared to LEAN such as DPT, ELN, FN1, SPARC, MFAP5, MGP, BGN and lysyl oxidase like 1 (LOXL1) were down-regulated in MASH treated group with 0.1 µM anti-TGF-β Ab. GSEA analysis revealed that many of the strongly up-regulated pro-fibrotic pathways in MASH hLiMTs including ABV3 integrin, integrin 1, and integrin cell surface interactions, non-integrin membrane ECM interactions, canonical and non-canonical TGF-β signaling, TGF-β receptor signaling, syndecan 1 and syndecan interactions, epithelial mesenchymal transition, extracellular matrix organization and proteoglycans, formation, assembly, crosslinking and trimerization of collagen fibers, degradation of ECM and collagen were down-regulated upon treatment with 0.1 µM anti-TGF-β Ab (Fig. 4F).

**Figure 5:**
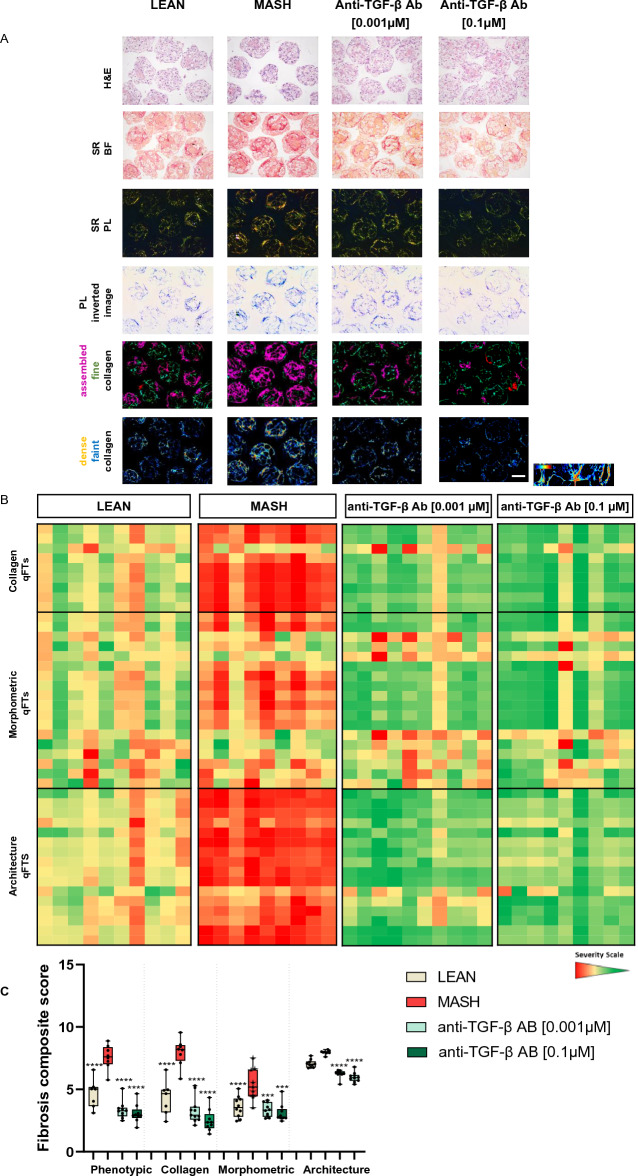
Anti-TGF-β Ab treatment decreases the deposition of fibrillated collagens in MASH hLiMTs. (**A**) H&E and SR staining of deposited fibrillated collagens (SR-BF and SR-PL images) and their quantification by FibroNest™ II platform of LEAN, MASH and MASH treated with 0.001 µM or 0.1 µM anti-TGF-β Ab hLiMTs on day 10. Green and purple are fine and assembled collagen, respectively. Blue and yellow/red are faint and dense collagen, respectively. The images are taken with 10 × magnification, scale bar: 100 µm. (**B**) Heatmap of the extent of fibrosis severity, ranging from green: faint and fine collagen to red: dense and complex collagen. Every column represents a hLiMT sample, and every row is a different quantitative fibrosis traits (qFTs) that contribute to the collagen, morphometric and architecture sub-phenotypes. (**C**) Ph-FCS, collagen-FCS, morphometric-FCS and architecture-FCS generated by 43 qFTs (Supplementary table 2). Mean ± SD, n ≥ 8 microtissues/group, *p ≤ 0.05, **p ≤ 0.01, ***p ≤ 0.001, ****p ≤ 0.0001, (one-way ANOVA, Welch's correction), MASH vs LEAN or compound treatment.

To investigate whether anti-TGF-β Ab can affect the deposition of fibrillated collagens, SR staining was performed on the tissue histology slices (Fig. 5). Compared to MASH hLiMTs, after treatment with 0.001 µM or 0.1 µM anti-TGF-β Ab, there was a decrease in the deposition of collagen fibrils visualized under BF and PL in histological sections stained with SR (Fig. 5A). The analysis of the SR stained tissues by FibroNest™ II software revealed that the deposition of collagen fibrils altered upon treatment with both concentrations of anti-TGF-β Ab. The IgG control did not significantly affect the deposition of collagens (data not shown). FibroNest™ II phenotypic fibrosis quantification of SR stained MASH hLiMTs sections indicated a significant reduction of all major features of fibrosis upon 0.001 µM or 0.1 µM anti-TGF-β Ab treatment. Anti-TGF-β Ab-treated hLiMTs exhibited reduced deposition of assembled and dense collagens (purple, yellow and red color fibers, respectively), which had been very pronounced in the MASH conditions (Fig. 5A). There was basal amount of deposition of fine and faint collagen fibers (green and blue color fibers, respectively) in all groups (Fig. 5A). Under the LEAN condition, where PBS serves as the vehicle control for anti-TGF-β Ab treatment, a lower level of fibrosis architecture qFTs was observed compared to the 0.2% DMSO vehicle control for ALK5i treatment (Figs. 3C and 5C). The quantification of fibrosis as a heatmap highlighted higher fibrosis in MASH compared to the LEAN and anti-TGF-β Ab-treated hLiMTs (Fig. 5B and Supplementary Table 2). Both concentrations of anti-TGF-β Ab exhibited an anti-fibrotic effect as shown by the decrease of Ph-FCS and its three sub-phenotypes (i.e. collagen-FCS, morphometric-FCS and architecture-FCS, Fig. 5C). Many qFTs were down-regulated upon anti-TGF-β Ab treatment associated with collagen content (e.g. skeleton notes and branches, total collagen area ratio, assembled collagen, total density area ratio), morphometry (e.g. total skeleton length, perimeter, area and width) and architecture (e.g. normalized count of uniform inertia, normalized count of contrasted inertia, mean and median inertia, kurtosis and skew) sub-phenotypes of fibrosis (Supplementary Table 2).

### Mechanistic investigation of the effect of clinical compounds Firsocostat and Selonsertib as single agents and in combination

#### Anti-fibrotic effect of Firsocostat and Selonsertib on phenotypically quantified fibrosis using AI FibroNest™ imaging platform

**Figure 6:**
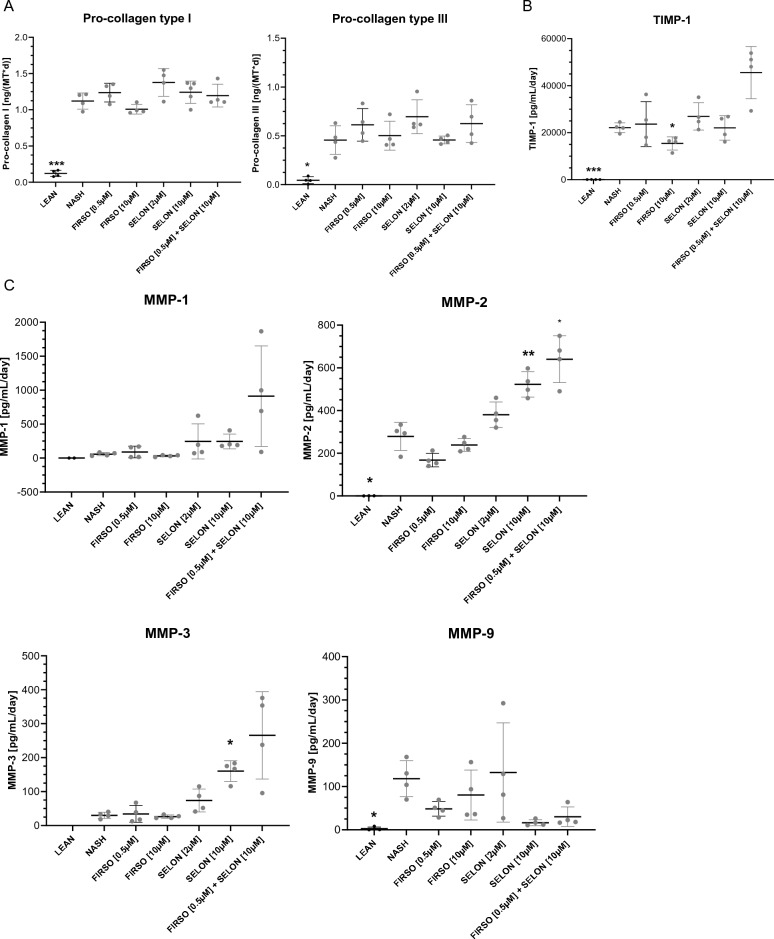
Firsocostat and Selonsertib change the secretion of pro-fibrotic markers. (**A**) Pro-collagen type I and pro-collagen type III (**B**) TIMP-1, (**C**) MMP-1, MMP-2, MMP-3 and MMP-9 in LEAN, MASH and MASH treated with 0.5 µM and 10 µM Firsocostat (FIRSO), 2 µM and 10 µM Selonsertib (SELON), 0.5 μM FIRSO with 10 μM SELON hLiMTs on day 10. Mean ± SD, n = 4 microtissues/group, data points below the lowest standard value were excluded from the data set. *p ≤ 0.05, **p ≤ 0.01, ***p ≤ 0.001, ****p ≤ 0.0001, (one-way ANOVA, Welch's correction), MASH vs LEAN or compound treatment.

There was no effect of both tested concentrations of Firsocostat and Selonsertib, alone or in combination, on the secretion of pro-collagen type I or III on day 10 (Fig. 6A). A concentration of 10 µM Firsocostat decreased significantly the secretion of clinical biomarker, TIMP-1, in contrast to Selonsertib, which did not have any effect (Fig. 6B). The effect of these clinical compounds on the secretion of TIMP-2 was not tested. A concentration of 10 µM Selonsertib significantly increased the secretion of anti-fibrotic MMP-2 and MMP-3. A combination of 0.5 μM Firsocostat with 10 μM Selonsertib and 10 μM Selonsertib demonstrated a tendency to decrease the secretion of pro-fibrotic MMP-9 (Fig. 6C).

**Figure 7:**
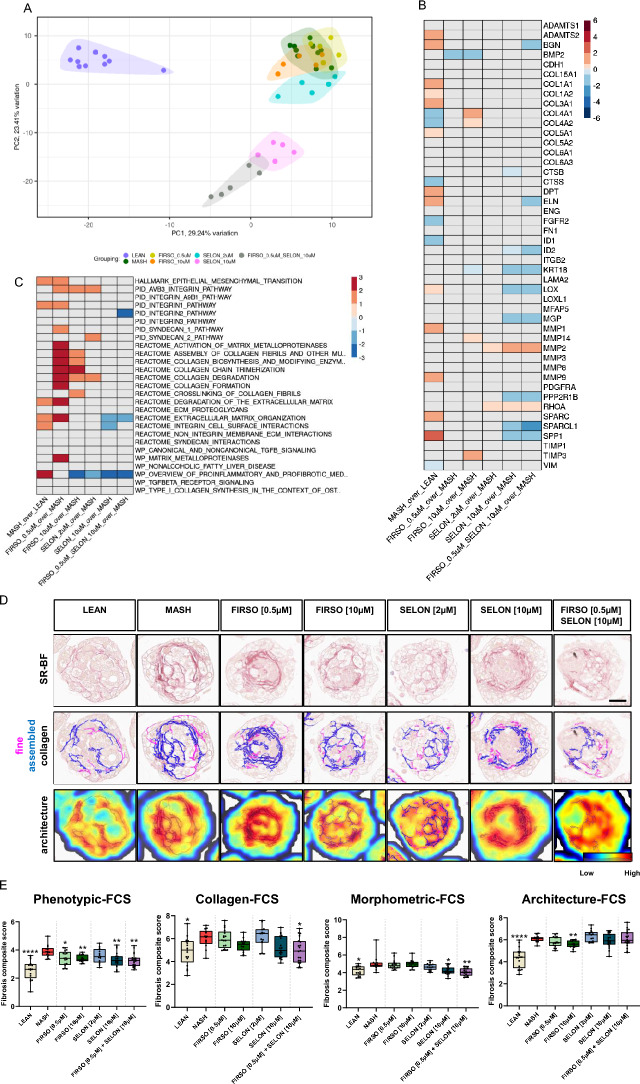
Firsocostat and Selonsertib decrease the fibrotic gene expression levels and the deposition of the fibrillated collagens. (**A**) PCA plot presenting intervention-wise-grouping of LEAN, MASH and MASH treated with FIRSO, SELON and FIRSO with SELON hLiMTs on day 10. (**B**) Summary heatmap presenting DEA results on day 10 for the selected genes related to fibrosis (Supplementary table 4) in MASH hLiMTs compared to LEAN controls (column 1) and in MASH hLiMTs treated with FIRSO, SELON and FIRSO with SELON compared to MASH counterparts (column 2–6). Color scale represents Log2FC values derived from DEA (Wald test, Benjamini–Hochberg correction). Log2FC values associated with FDR > 0.01 were overwritten with zero and are depicted in grey. Color scale range was determined based on the spread of the log2FC values for all genes investigated in the study. (**C**) Summary heatmap presenting GSEA results on day 10 for the selected gene sets related to fibrosis (Supplementary table 5) in MASH hLiMTs compared to LEAN controls (column 1) and in MASH hLiMTs treated with FIRSO, SELON and FIRSO with SELON compared to MASH counterparts (column 2–6). Color scale represents NES values derived from GSEA (Kolmogorov–Smirnov-like statistic + permutation test, Benjamini–Hochberg correction). NES values associated with FDR > 0.05 were overwritten with zero and are depicted in grey. Color scale range was determined based on the spread of the NES values for all gene sets investigated in the study. (**D**) SR-BF images and their quantification by FibroNest™ III image platform of LEAN, MASH and MASH treated with FIRSO, SELON and FIRSO with SELON hLiMTs on day 10. Purple is fine collagen fibers and blue is assembled and highly reticulated collagen fibers. The scan images are taken with 40 × magnification, scale bar: 50 µm. (**E**) Ph-FCS, collagen-FCS, morphometric-FCS and architecture-FCS generated by 206 qFTs. Mean ± SD, n ≥ 11 microtissues/group, *p ≤ 0.05, **p ≤ 0.01, ****p ≤ 0.0001, (one-way ANOVA, Welch's correction), MASH vs LEAN or compound treatment.

We performed whole transcriptomic profiling of MASH hLiMTs treated with each of these clinical compounds alone or in combination on day 10 (Fig. 7). PC1 and PC2 plots revealed clustering of the LEAN away from the MASH group (Fig. 7A). The 2 µM Selonsertib, 0.5 µM and 10 µM Firsocostat treated groups clustered near to the MASH group, whereas the 10 µM Selonsertib and combined 0.5 µM Firsocostat with 10 µM Selonsertib treated groups clustered separately from the MASH and other treated groups (Fig. 7A). The change in gene expression by 10 µM Selonsertib was similar to that by 0.5 µM Firsocostat with 10 µM Selonsertib (Fig. 7B). These treatments upregulated the gene expression of anti-fibrotic MMP-2 but down-regulated the expression of the pro-fibrotic markers SPP1, secreted protein, acidic and rich in cysteine-like 1 (SPARCL1), MGP, keratin 18 (KRT18), protein phosphatase 2 scaffold subunit A beta (PPP2R1B) and lysyl oxidase (LOX). A synergistic effect of 0.5 µM Firsocostat with 10 µM Selonsertib resulted in decreased expression of ELN, BGN, SPARCL1 and inhibitor of DNA binding 2 (ID2). The GSEA analysis indicated that Firsocostat upregulates pathways related to MMPs, ECM organization and degradation, collagen degradation, biosynthesis and formation, assembly of collagen fibrils, integrin 1, syndecan 1 and ABV3 integrin. The combination of 0.5 µM Firsocostat with 10 µM Selonsertib treatment resulted in down-regulation of integrin 2, extracellular matrix organization, pro-inflammatory and pro-fibrotic pathways (Fig. 7C).

Next generation high sensitivity FibroNest™ III software was used to analyze MASH hLiMTs treated with both clinical compounds as mono and combinatorial treatment. These results showed that the clinical compounds caused a decrease in the deposition of collagen fibrils, visualized under BF and PL on histological sections stained with SR (Fig. 7D and Supplementary Fig. 1). The quantification of fibrosis demonstrated again a substantial increase in fibrosis in the MASH conditions compared to the LEAN control as seen in the Ph-FCS and its sub-phenotypes (Fig. 7E). Firsocostat and Selonsertib decreased the formation of assembled and dense collagens (blue color fibers, Fig. 7D), which had been very pronounced in the MASH conditions. In LEAN hLiMTs there was basal level of deposition of fine collagen fibers with minimal branching and nodes (purple color fibers) as well as little formation of assembled collagen (blue color fibers, Fig. 7D and Supplementary Fig. 1) as compared to MASH conditions. The data from quantification of fibrosis are presented as a Ph-FCS and a heatmap, which offers a significant detection threshold and dynamic range to evaluate the anti-fibrotic responses of the treatment groups (Fig. 7E and Supplementary Table 3). Both concentrations of Firsocostat (0.5 and 10 µM) and the higher concentration of Selonsertib (10 µM) exhibited anti-fibrotic effects as shown for the Ph-FCS. However, the effect of these two compounds alone on the sub-phenotypes is quite variable. Interestingly, the combination of 0.5 µM Firsocostat with 10 µM Selonsertib did show anti-fibrotic effects on Ph-FCS and its sub-phenotypes, except for the architecture-FCS.

## Discussion

Here, we present MASH hLiMT model that uses spheroidal, scaffold free co-cultures of primary human hepatocytes, Kupffer cells, liver endothelial cells and hepatic stellate cells. The cells are cultured in 96-well plates coated with a non-adhesive surface solution preventing tissues adherence to the plastic. The cells self-assemble to form tissues, thus promoting cell–cell and cell–matrix interactions. The hLiMTs generate their own optimal ECM; therefore, an external in vivo-like matrix is not required to generate this MASH model. Upon exposure to clinically relevant lipotoxic and inflammatory stimuli, these microtissues develop key pathophysiological features of MASH within 10 days, including an increase of tissue triglyceride levels, release of pro-inflammatory markers, synthesis of collagen type I/III and deposition of fibrillar collagens^13^**.**

MASH is a progressive and severe liver disease characterized by lipid accumulation, inflammation and, later, fibrosis. The different stages in the development of MASH occur over decades and involve a complex interaction of multiple cell types^3^. Therefore, the efficacy testing of anti-fibrotic compounds should be based on multiple endpoints to be able to detect different attributes of the pathology and the pathways involved. The endpoints range from functional assays such as secretion of pro-collagen type I and III peptides, TIMPs and MMPs, over gene expression analysis to histological assessments of fibrosis deposition. These endpoints provide different information related to fibrosis. The secretion of pro-collagen type I and III peptides reflects the effect of compounds on the new synthesis of collagens and the development of fibrotic process^13^; the secretion of MMPs and TIMPs are clinical biomarkers for MASH and relate to the degradation of extracellular matrix^32–34^. SR staining to visualize collagen fibrillation and deposition is a well-established method to qualify pathological changes of collagen fiber content and structure in clinical tissues sections^13,35^. An evaluation of the histology of liver biopsies is still the gold standard technique for assessing fibrosis in MASH patients despite its shortcomings due to patient discomfort, sampling variability, intra- and inter-observer variability, cost, etc.^20^. In the past decade, there has been an increasing interest in the use of NITs such as ELF test, liver elastography (FibroScan®), FIB-4 index, etc. in the clinic for assessment of MASH and fibrosis scoring in patients. Histological analysis of MASH may be superseded by NITs in the future, but in the meantime, the development and validation of DP/AI tools for phenotypic fibrosis quantification should be of high priority in the development of MASH drugs. Multiple companies are developing different DP/AI tools; therefore, it is crucial to determine in which patients or studies the different tools are most effective for analysis of clinical data sets. Furthermore the same or similar DP/AI tools for assessing the effect of compounds should be conducted in both pre-clinical and clinical studies to achieve maximum translation of the results through the development process^36^.

The development of novel anti-fibrotic therapies has been hindered, in part, by the limitations of existing fibrosis analysis techniques of the histology samples from in vivo and in vitro preclinical models. We have reported previously the performance of novel quantitative DP/AI platform, FibroNest™, to generate automatic, continuous, and direct fibrosis endpoints to quantify fibrosis severity and compound treatment response in clinical MASH samples^23^. The aim of the current work was to establish a novel algorithm for automated quantification of fibrosis in an in vitro MASH hLiMT model based on the FibroNest™ platform used to quantify fibrosis in clinical samples^22,35^. The new algorithm for the phenotypic quantification of fibrosis was adapted from clinical biopsy samples and aimed to complement the multiparametric analysis of MASH hLiMTs. The initial quantification of fibrosis after treatment with the reference anti-fibrotic compounds, ALK5i and anti-TGF-β Ab, was conducted using FibroNest™ II platform. The quantification of fibrosis after treatment with the clinical compounds Firsocostat and Selonsertib was conducted using more sensitive version FibroNest™ III software, which better detects even finer deposited collagen fibers using specific algorithm (Fig. 7D, E, Supplementary Fig. 1 and Supplementary Table 3). The results of the FibroNest™ image analysis were compared with the functional and gene expression endpoints.

We have established several biochemical assays for the detection of fibrosis, including the secretion of clinical biomarkers such as collagen type III and TIMP-1, which are part of the of ELF kit used extensively in clinical trials as a noninvasive method for assessment of activity and dynamic of fibrosis^37^. Treatment of the hLiMTs with sugars, insulin, FFAs and LPS induced a phenotype similar to that observed in human MASH patients^38^. The secretion of pro-collagen type I and III by MASH hLiMTs was 8–10-fold higher than LEAN hLiMTs, which is in line with the excessive amount of these fibrillar extracellular matrix proteins, collagen I and III, in the space of Disse in patients with liver fibrosis^39^. The gene expression analysis demonstrated up-regulation of the fibrillar collagens, such as COL1A1, COL1A2, COL3A1, COL5A1 and COL5A2, as well as non-fibrillar collagens COL6A1, COL6A3 and COL15A1 in MASH vs LEAN hLIMTs (Figs. 2E, 4E and 7B). The basement membrane is formed of collagen type IV (col IV), laminin, nidogen (NID) and perlecan, as well as some minor proteins such as fibronectin and collagen type XVIII (col XVIII). Elevated serum col IV and laminin can be used as biomarkers of chronic liver diseases and the severity of fibrosis. The fibrillar collagens genes such as COL4A1 and COL4A2, which are part of basement membrane, were down-regulated (Figs. 2E and 7B) or not changed (Fig. 4E) in MASH vs LEAN hLiMTs. The basement membrane molecules are produced by HSC, sinusoidal endothelial cells and biliary epithelial cells. The cellular sources of col XVIII are hepatocytes and HSC^40^. Healthy hepatocytes do not produce basement membrane proteins, but do so following chronic injury and fibrosis^41^. The basement membrane genes such as LAMA2, FN1 and HSPG2^40^ were up-regulated in MASH vs LEAN hLiMTs conditions (Figs. 2E, 4E and 7B). Other basement membrane genes, such as COL4A3-COL4A6, COL18A1 and NID1, were not significantly changed in MASH vs LEAN hLIMTs conditions (data not shown). It is important to further investigate the protein expression and secretion of basement membrane markers by Luminex multiplex technology or ELISA. The secretion of MMP-1, -2, -3 and -9 and TIMP-1 and -2, were also increased in MASH conditions compared to LEAN hLiMTs. MMP-1, secreted by activated HSC is known to enhance tissue fibrosis in MASH, suggesting it contributes to the repair and regeneration of the liver^42^. The mechanism of action of MMP-2 is mainly by changing the degree of collagen deposition during activation of liver HSC, rather than having a direct impact on the breakdown of collagen^43^. MMP-2 secreted by activated HSC, can degrade basement membrane molecules col IV and laminin, similar to MMP-9 secreted by KC and inflammatory macrophages^40^. MMP-3 secreted by activated HSC, KC and inflammatory macrophages degrades matrix components (collagens, elastin, proteoglycans, fibronectin, and laminin) and activates other MMPs (MMP-1, MMP-7 and MMP-9)^42^. While some MMPs are anti-fibrotic (e.g., MMP-1, -2 and -3), others can have pro-fibrotic functions (MMP-9, -12 and -19)^43^. Similar to the functional assays, gene expression analyses have shown an increased expression of anti-fibrotic MMP-3 and pro-fibrotic MMP-9 genes in MASH hLiMTs compared to LEAN conditions (Fig. 2E). Other pro-fibrotic and inflammatory genes, such as SPP1, was also upregulated in MASH compared to LEAN hLiMTs, similar to its increased gene expression in MASH patients^44^. Increased DPT gene expression has been detected in MASH hLiMTs, which has been shown to correlate with ECM formation and fibrosis severity in MASH patients^44^ (Figs. 2E, 4E and 7B). The increased gene expression of potential biomarker BGN has been detected in MASH hLiMTs, which has been shown to regulate the ECM deposition and HSC activation through an heat shock protein 47-dependent mechanism^45^. ELN has been found to be up-regulated in MASH compared to LEAN hLiMTs, suggesting an establishment of advanced fibrosis in MASH hLiMTs as the elastic fibers are associated with formation of F3 stage of fibrosis^20^.

FibroNest™ has been used to investigate liver fibrotic conditions in both patients and animal models with MASH. In healthy liver biopsy there is a presence of basal perisinusoidal fine collagens. This low level of perisinusoidal fine collagens is greatly amplified in MASH conditions, resulting in not only an increase in fine collagen but also a coalesce of fine into increased assembled collagen. In early stages of fibrosis F1/F2 (mild/middle fibrosis) an increase in centrilobular perisinusoidal and periportal fine collagen that have thicken in width and length as compared to healthy livers was observed, which leads to moderate increase in assembled collagen. In late stages of fibrosis F3/F4 (advanced fibrosis/cirrhosis), although there are new fine collagens being produced, the old collagen is present as assembled collagen. This can be displayed as partially connected less-developed bridging septa and well-developed thick fibrous cirrhotic septa. FibroNest™ image analysis showed that there was an increased deposition of assembled and dense collagens and fibrillation in MASH compared to LEAN hLiMTs, whereby the fine collagens had developed into larger structures which coalesced to form assembled and complex (highly reticulated) structures similar to that found in patients with F2-F3 stage fibrosis^46^. Interestingly LOX and LOXL1 genes involved in fibrillar collagens and elastin cross-linking were up-regulated in MASH vs LEAN hLiMTs conditions (Figs. 4E and 7B). The cross-linking of ECM is strongly implicated in fibrosis progression and resistance to fibrosis reversal^47^**.** Secreted cross-linked pro-peptides of collagen type III can be measured by PC3X assay, which can be used as diagnostic and prognostic biomarker of advanced fibrosis, cirrhosis and hepatocellular carcinoma^48^**.** Future experiments can be performed to determine the levels of secreted cross-linked pro-peptides of collagen type III in the MASH hLiMTs and upon compound treatment. FibroNest™ platform can indirectly measure crosslinked fibrillar collagens. The fiber with the more intense SR stain has more packed microfibrils and thus the cross-linked triple helices subunits. FibroNest™ can measure the intensity of the SR staining of these collagen fibers and the changes between treatment groups through image analysis. This quantification is measured in the collagen density area ratio, assembled tissue collagen content and other fibrosis qFTs displayed in the phenotypic heatmaps (Supplementary Tables 1, 2 and 3).

Initial experiments evaluated the impact of different modality reference anti-fibrotic compounds, namely, a small molecule (ALK5i) and biologic (anti-TGF-β Ab antibody) on the panel of endpoints. ALK5i was used to block the TGF-β signaling by inhibiting TGF-β type I receptor kinase and preventing the activation of HSC and fibrosis^49^. The concentration of 0.5 µM ALK5i was shown previously to be effective in this model based on the secretion of pro-collagen type I^13^. Anti-TGF-β Ab inhibits soluble TGF-β -1, -2 and -3^50,51^, major pro-fibrotic mediators that activate quiescent HSC to a myofibroblast phenotype, which then produce fibrillary collagens such as collagen type I and III^39^. Anti-TGF-β Ab is therefore used widely as a tool to inhibit fibrosis. We observed that both reference compounds decreased the secretion of pro-collagen I and III, as well as MMPs and TIMPs, and these data aligned very well with the gene expression data with respect to differentially expressed genes and the inactivation of pro-fibrotic pathways. Treatment with 0.5 µM ALK5i or 0.1 µM anti-TGF-β Ab of MASH hLiMTs decreased the gene expression level of pro-fibrotic genes COL1A1, COL1A2, COL3A1, COL5A1, ELN, MMP-3, MMP-9, DPT, MGP, LOXL1, MFAP5, SPARC and BGN (Figs. 2E, 4E and 7B).

There was a pronounced anti-fibrotic effect of 0.001 µM anti-TGF-β Ab, evident as a strong inhibition of the secretion of pro-collagen type I, TIMP-1 and TIMP-2 similar to that caused by 0.1 µM, whereas effects of 0.001 µM on pro-collagen type III and MMP-3 secretion were much weaker than by 0.1 µM (Fig. 4A–C). This discrepancy might be explained by the stronger preventive effect of the anti-TGF-β Ab on the deposition, degradation and formation of assembled collagens than on synthesis of these proteins. Unlike anti-TGF-β Ab , ALK5i decreased the secretion of only TIMP-1 on day 10, but not TIMP-2; however, it did decrease the secretion of MMP-1, MMP-3, pro-collagen type I/III and the gene expression of other key fibrotic markers, such as COL1A1, COL1A2, COL3A1, COL5A1 and COL15A1 (Fig. 2A–C and E).

Both ALK5i and anti-TGF-β Ab treatments have shown a trend to increase MMP-9 secretion compared to the MASH hLiMT condition, however this effect was not statistically significant (Figs. 2C and 4C). By contrast, MMP-9 gene expression was down-regulated by ALK5i and not altered by anti-TGF-β Ab treatment (Figs. 2E and 4E). The differential effect of both compounds on protein secretion and gene expression of MMP-9 at given time can be related to negative feedback regulation. Importantly MMP-9 synthesis has been shown to be regulated by several mechanisms, including gene expression, post-transcriptional processing and post-translational level^52^. Dephosphorylation of extracellular signal-regulated kinase 1/2 (ERK1/2) has been shown to down-regulate MMP-9 mRNA expression in macrophages treated with cannabinoid agonist R( +) WIN55,212–2. However ERK 1/2 signaling has been shown not to affect the protein expression and secretion of MMP-9^53^. To demonstrate the direct effect of ALK5i and anti-TGF-β Ab on fibrosis degradation, the activities of different MMPs and the formation of degraded collagen type I and III fragments should be assessed.

The quantification of fibrosis using FibroNest™ II analysis indicated a decrease in phenotypic-, collagen-, morphometric- and architecture-FCS after treatment with both reference compounds (Figs. 3C and 5C). These findings indicate that the functional and gene expression data correlated with histological quantification of fibrosis with respect to anti-fibrotic effect of these reference compounds. Importantly, the fibrosis architecture qFTs were increased in LEAN hLiMTs by 0.2% DMSO vehicle control for ALK5i, whereas PBS vehicle control for anti-TGF-β Ab did not show such an effect (Figs. 3C and 5C). Increased basal fibrosis was observed when the DMSO was used at concentrations ≥ 0.5% (data not shown). Therefore, the concentration of DMSO was kept at 0.2% for the efficacy tests of small molecules to ensure only a slight increase of fibrosis architecture qFTs due to the solvent. Although 0.2% DMSO alters the basal fibrosis architecture qFTs, there were still marked fold changes between MASH vs LEAN hLiMTs conditions based on observations of all the measured biomarkers and Ph-FCS.

When the anti-fibrotic effects of Firsocostat and Selonsertib were evaluated using the same panel of endpoints, there was a smaller compounds effect on the functional assays and gene-expression as compared to the phenotypic quantification of fibrosis using FibroNest™ III image analysis. Firsocostat and Selonsertib treatment alone or in combination did not decrease the secretion of pro-collagen type I and III (Fig. 6A). Anti-fibrotic MMP-2 and -3 secretion was increased by 10 µM Selonsertib treatment alone or in combination with 0.5 µM Firsocostat (Fig. 6C). The clinical biomarker TIMP-1 secretion was decreased by 10 µM Firsocostat (Fig. 6B).

Firsocostat, an ACC1 and 2 inhibitor (ACCi), has been shown to reduce hepatic fat, lipogenesis and liver injury markers in patients with MASH^28,54,55^. Firoscostat modulates mitochondrial fatty acid oxidation involved in de novo lipogenesis. An increase in lipogenesis and reduced fatty acid oxidation are thought to contribute to steatosis. Inhibition of ACC and de novo lipogenesis are reported to repress the activation of HSC and thus leading to less fibrosis^56,57^. ACCi has been shown to reduce liver fibrosis by both reducing lipotoxicity in hepatocytes and directly reducing HSC activation, as measured by both α-SMA expression and collagen production. The lack of a significant effect of both 0.5 µM and 10 µM of Firsocostat on secreted pro-collagens I and III indicates that its anti-fibrotic activity is not related to the collagen synthesis. A concentration of 10 µM Firsocostat decreases TIMP-1 secretion, demonstrating its anti-fibrotic effect on the collagen degradation and ECM remodeling (Fig. 6B). The GSEA results have shown that Firsocostat upregulates pathways related to activation of MMPs, biosynthesis, formation and degradation of ECM as well as collagen. The combinatorial treatment of Firsocostat and Selonsertib resulted in down-regulated integrin, extracellular matrix organization and pro-inflammatory and pro-fibrotic pathways (Fig. 7C). In line with these results, the combinatorial Firsocostat and Selonsertib treatment up-regulated the gene expression of anti-fibrotic MMP-2 but down-regulated the expression of the pro-fibrotic markers such as SPP1, ELN, BGN, KRT18, ID2, MGP, SPARCL1, PPP2R1B and LOX^20,33^ (Fig. 7B). A more sensitive analysis of the deposition of fibrillated collagen fibers on day 10 using FibroNest™ III imaging platform indicated that both concentrations of Firsocostat exhibited anti-fibrotic effects (Fig. 7E). Our previous published work has shown that 0.5 μM and 10 μM Firsocostat decrease the tissues triglyceride levels in MASH hLiMTs but has no effect on secretion of pro-inflammatory markers and only tendency to decrease the pro-collagen type I^13^.

ASK1 is activated in oxidative stress, causing inflammation and apoptosis which leads to liver fibrogenesis^29^. The anti-fibrotic effect of the ASK1 inhibitor, Selonsertib, (10 µM) in hLiMTs was evident as a tendency to decrease the pro-fibrotic MMP-9 and increase of anti-fibrotic MMP-2 and MMP-3 protein secretion and MMP-2 gene expression (Figs. 6C and 7B). Therefore, the changes in MMP gene expression and protein secretion supports ASK1 anti-fibrotic action. Selonsertib is reported to reverse fibrotic induction by decreasing HSC activation and proinflammatory responses^14^. In accordance with this and clinical findings^58^, the effects of Selonsertib in this study were evident as a decrease in the pro-inflammatory pathways, and pro-fibrotic pathways such as integrin cell surface interaction and extracellular matrix organization (Fig. 7C). Anti-inflammatory activity of Selonsertib was reflected as a concentration-dependent decrease in the secretion of pro-inflammatory cytokines: TNF-α and IL-6 and chemokines: MCP-1, MIP-1α, IL-8, IP-10 in our previously published work^13^. Selonsertib improved fibrosis in the treatment of MASH in Phase II clinical trials after its administration for 24-weeks in patients with moderate to severe fibrosis F2/F3^30^. However, its further development has been stopped due the failure to improve fibrosis without worsening of MASH in phase III clinical trials, in which Selonsertib was given for 48-weeks in MASH patients with bridging fibrosis F3 or compensated cirrhosis F4^1,31^. Based on the Ph-FCS and morphometric-FCS using FibroNest™ image analysis platform, 10 µM Selonsertib was shown to exhibit anti-fibrotic effects in line with the results of Phase II clinical trial^1^. However, while this combination of Firsocostat and Selonsertib was well-tolerated, it did not meet the primary endpoint of reducing fibrosis without worsening of MASH. The lack of enhanced efficacy of the combination of Firsocostat and Selonsertib was mirrored by the functional and FibroNest™ results, which showed no synergistic anti-fibrotic effects.

## Conclusions

We have established a novel single-fiber AI algorithm for automatic quantification of fibrosis using FibroNest™ image analysis platform in MASH hLiMTs based on the model used for quantifying fibrosis in clinical samples. The FibroNest™ DP/AI platform provides accurate and precise approach for quantitative assessment of changes on a continues scale detecting subtle nuances and characteristics of deposited collagen fibers, as well as characterizing spatial and architectural changes in MASH hLiMTs. The results obtained from FibroNest™ were in good accordance with the outcome of the secretion of pro-fibrotic biomarkers of anti-fibrotic reference compounds, thus validating this tool. This study indicated that the secretion of the fibrotic biomarkers and transcriptomics are not sufficient to assess the anti-fibrotic mechanism of action of clinical compounds and that they should be supplemented with the gold standard techniques such as SR staining of histology slides and their fibrosis quantification, including multiple qFTs. The traits distributed among three sub-phenotypes (collagen structure, morphometry and architecture) can give further insight into the mechanism of action of anti-fibrotic compounds. This study has also shown that MASH hLiMT model can be used for efficacy assessment of different modalities drug-candidates like small molecules and antibodies.

In conclusion, we demonstrated that combining FibroNest™ image analysis for phenotypic quantification of deposition of collagen fibers with secretion of pro-fibrotic markers and transcriptomics in MASH hLiMTs can provide a powerful high throughput platform to evaluate comprehensively anti-fibrotic properties of novel drug candidates in pre-clinical studies. This approach might improve the results of the preclinical efficacy studies of anti-fibrotic compounds and their translational value to the clinical trials.

## Methods

### Liver microtissue maintenance and disease induction

hLiMTs were comprised of a pool of PHH from 10 donors, single-donor HSC and single-donor KC and LEC (MT-02–302-05, InSphero AG). The informed consent of the human liver cells for all the subjects was obtained from the vendor. hLiMTs were generated by self-assembly of monodispersed primary cells, as described previously^18,59,60^. hLiMTs were maintained at 37 °C, 5% CO_2_ and 95% humidity. To mimic MASH disease induction and progression, hLiMTs were exposed for 10 days to either LEAN or MASH conditions (Fig. 1B). LEAN condition hLiMTs were cultured in basal hepatocyte maintenance medium (CS-07–302-01, InSphero AG). Under disease conditions, the MASH induction medium was supplemented with elevated insulin amount, glucose and fructose levels (total of 22.5 mM) (CS-07–301-01, InSphero AG), FFAs (167 µM, CP-02–302, CP-02–303, InSphero AG), and LPS (5 µg/ml LPS, Sigma-Aldrich). LPS was applied as a pulsing on day 3 of treatment. Medium was exchanged on days 0, 3, 5, and 7.

### Compound treatment

The anti-fibrotic effects of reference compounds, 0.5 µM ALK5i (SB525334, Selleckchem) and 0.001 and 0.1 µM anti-TGF-β Ab (MAB1835, R&D Systems), were investigated by incubating hLiMTs in MASH induction medium containing high levels of sugars, insulin, FFAs and LPS pulse in the presence or absence of the drug (Fig. 1B). Compound treatments were performed on days 0, 3, 5 and 7, using a TECAN D300e Digital Dispenser or manually for the anti-TGF-β Ab. All conditions were normalized to vehicle controls 0.2% DMSO or PBS (only for anti-TGF-β Ab).

For the efficacy test of anti-MASH clinical compounds an optimized 3D MASH model was used. This optimized 3D MASH model was generated using the improved production protocol of the microtissues to achieve reduced levels of secretion of pro-collagen type I and tissue triglyceride levels in the LEAN conditions. The clinical test compounds were Firsocostat (HY-16901, MedChemExpress) and Selonsertib (HY-18938, MedChemExpress). These and the reference compound, ALK5i and anti-TGF-β Ab, were applied during the 10-day MASH induction protocol. The final concentrations were 0.5 µM ALK5i, 0.001 µM and 0.1 µM anti-TGF-β Ab, 0.5 µM and 10 µM Firsocostat, 2 µM and 10 µM Selonsertib, and a combination of 10 μM Selonsertib and 0.5 μM Firsocostat.

All stock solutions were prepared in DMSO except for anti-TGF-β Ab stock, which was prepared in PBS. The anti-fibrotic effects of the tested compounds were assessed according to several endpoint measurements described below.

### Endpoint assays

All assays were performed with 4 to 6 biological replicates per treatment group and were repeated at least twice. For all experiments the same lots of PHH, KC, LEC and HSC were used.

#### Pro-collagen I secretion measurement

The levels of cleaved and secreted C-terminal human collagen type I pro-peptide in hLiMT supernatants collected from day 10 were measured with the human pro-collagen I homogeneous time-resolved fluorescence (HTRF®) assay (Cisbio). PBS (Sigma-Aldrich) diluted samples were processed according to the supplier’s instructions. Fluorescence was measured with a Tecan Spark 10M plate reader. Pro-collagen I secretion rate per hLiMT was calculated by linear fit to a standard curve. Values normalized to incubation time (days) were plotted as mean ± SD, including individual datapoints. A Brown-Forsythe version of one-way ANOVA (analysis of variance) with Welch’s correction in combination with Dunnett’s T3 multiple comparisons test vs MASH was used to calculate significant changes.

#### Pro-collagen III secretion measurement

The levels of cleaved and secreted N-terminal human collagen type III pro-peptide in hLiMT supernatants collected from day 10 were measured with the human N-Terminal Pro-collagen III immuno-assay (P3NP-ELISA, Cisbio). PBS diluted samples were processed according to the supplier’s instructions. Optical density (OD) at 450nm was measured with a Tecan Spark 10M plate reader. Pro-collagen III pro-peptide secretion per hLiMT was calculated by 4-parameters logistic (4-PL) mathematical fit curve. Interpolated data points below the lowest standard value were excluded from the data set. Values normalized to treatment time were plotted as mean ± SD, including individual datapoints. A Brown-Forsythe version of one-way ANOVA with Welch’s correction in combination with Dunnett’s T3 multiple comparisons test vs MASH was used to calculate significant changes.

#### Pro-fibrotic markers secretion

Pro-fibrotic markers TIMP-1, TIMP-2, MMP-1, MMP-2, MMP-3 and MMP-9 in cell supernatants were determined on day 10 using the Magnetic Luminex® Performance Assay:  Human TIMP Multiplex Kit, LKTM003, TIMP-1 and -2 (premixed, Bio-Techne), Human MMP Multiplex Kit, LXSAHM-07 (Bio-Techne) and customized ProcartaPlex™, PPX-06 (MMP-1, -2, -3, -9, TIMP-1, eBioscience). The multiplexed assay was performed according to supplier’s instructions with minor adaptation of microparticle and Ab concentrations to analyte levels present in supernatants. Measurements were performed with a Luminex® MAGPIX® analyzer. Protein concentrations were calculated by 5 parameter logistic fit (5-PL) to a standard curve using Luminex XPonent software. Interpolated data points below the lowest standard value were excluded from the data set. Data are represented as mean values ± SD, including individual datapoints. A Brown-Forsythe version of one-way ANOVA with Welch’s correction vs MASH in combination with Dunnett’s T3 multiple comparisons test vs MASH was used to calculate significant changes. Outliers were detected based on Grubbs’ test (α = 0.05).

#### RNA sequencing (RNA-Seq) and analysis

RNA-Seq in the study was performed by using whole transcriptome TempO-Seq® assay consisting of 22,537 probes targeting 19,701 genes (BioSpyder Technologies, Inc.)^61^. TempO-Seq samples were generated as follows: upon experiment completion on day 10 of treatment, single hLiMTs were washed in PBS without Ca2 + /Mg2 + and lysed in 15 µl of 1 × Enhanced Lysis Buffer (BioSpyder Technologies, Inc.). Libraries generation from crude lysates and single-end 50 bp sequencing on Illumina HiSeq 2500 platform were performed by BioSpyder Technologies, Inc. After sample demultiplexing and obtaining sample-wise FASTQ files, reads alignment and counting were performed using TempO-SeqR data analysis tool (BioSpyder Technologies, Inc.). The gene expression data were then analyzed with the InSphero RNA-Seq analysis pipeline. In brief, the probe-wise raw count table was collapsed toward the gene-wise count table by summating counts for probes associated with the same gene. Next, the gene-wise count table was cleaned from genes that were not detected or very lowly expressed. Subsequently, data normalization, principal component analysis (PCA) and differential expression analysis (DEA) were performed as implemented in the DESeq2 R package^62^. Importantly, surrogate variable analysis (SVA)^63^ was applied to remove unintended batch effects by using the sva R package. Furthermore, the pre-ranked type of GSEA^64^ was performed using the clusterProfiler R library^60,65^. In GSEA, genes were pre-ranked by log2FC values derived from DEA and normalized enrichment score (NES) was used as an enrichment metric. The following resources from Molecular Signatures Database (MSigDB)^66^ version 7.5 were used to build a repertoire of gene sets queried in GSEA: BioCarta [BioCarta^67^], Hallmark^66^, PID^68^, Reactome^69^ and WikiPathways^70^, in total 2817 gene sets. For both, DEA and GSEA, the Benjamini–Hochberg procedure for false discovery rate (FDR)^71^ was used to correct p values across contrasts, and the FDR cut-offs were set to 0.01 and 0.05, respectively.

#### Formalin-fixation and paraffin-embedding (FFPE) of hLiMTs

On day 10 of treatment, > 18 hLiMTs per treatment group were pooled, washed with PBS, and fixed with 4% paraformaldehyde (PFA, Alfa Aesar) for 1 h at RT. The fixed hLiMTs were washed in PBS, pelleted in 1.7% agarose, and further processed with a tissue processor (Logos Microwave Hybrid Tissue Processor; Milestone Medical S.r.I) for paraffin embedding. The paraffin blocks were sectioned to 4 µm thickness using a microtome (Histocore Multicut, semimotorized Rotation Microtome; Leica Biosystems). The sections were mounted on poly-L lysin treated glass slides (HistoBond® + S with grounded edge; Marienfeld) and dried for 60 min at 65°C.

#### Immunohistological (IHC) and SR staining

Hematoxylin (Mayers Hematoxylin, Leica Biosystems) and eosin (eosin 2% aqueous, Chroma Waldeck) staining was performed as described previously^72^. For visualization of fibrillated collagen structures, a SR staining procedure^73^ with slight modifications, was applied. After rehydration, the sections were stained with SR solution (SR solution, Chroma Waldeck) for 5 min and stopped with 0.5% acidic acid solution (without hematoxylin counterstaining).

#### Quantification of fibrosis using SR-stained tissues

##### Digital image analysis and fibrosis quantification

SR-stained sections images of the treated tissues with ALK5i and anti-TGF-β Ab were captured using a Leica DMi8 microscope at 10x (HC PL FLUOTAR 10x/0.32 PH1) or 20x (HC PL FL L 20x/0.40 CORR PH1) magnification. Automated white balance correction was applied, and images were captured with a DMC4500 digital camera. For the clinical compounds treatments, the whole slides with SR stained sections were digitally scanned in an Aperio GT 450 DX system (Leica Biosystems Inc.) at 40 × (0.221 μm per pixel). Automated white balance correction was applied, and images were captured with a DMC4500 digital camera. For the PL images, a motorized, polarized filter was used. Images were inverted using ImageJ invert function for better visualization. The images were saved as svs or TIFF files. FibroNest™ image analysis platform was used to quantify the fibrosis in these MASH hLiMT slices. This analysis had several steps, outlined here.

##### Microtissue adequacy

Each microtissue was evaluated for quality by size. Microtissues which were too small (< 50% of mean microtissue area) were excluded from the study.

##### Fiber detection

FibroNest™ was used for the quantification of SR- positive stained collagen fibers from each image. After deconvolution (an algorithm-based image processing to extract collagens stained with SR) and selection of adequate microtissues for analysis, FibroNest™ was used to detect each collagen fiber in a stained digital histology image (Fig. 1A). The architecture (i.e., the organization and the complex buildup of multiple fibers) was measured by dividing the image into “computational windows” of 25 μm × 25 μm and then the fibrosis phenotype quantified in each window.

##### Analysis of fibers

FibroNest™ II image analysis software was used previously for the quantification of fibrosis in clinical and preclinical MASH tissues generating more than 300 phenotypic qFTs, which are organized into 3 different sub-phenotypes including collagen content, morphometry, and architecture^21–25,28^. Ph-FCS is a multifactorial and continuous score to quantify the phenotype of fibrosis, including collagen content, fibers morphometrics, and fibrosis architecture. Collagen-FCS is a multifactorial and continuous score for tissue collagen content with a focus on its general properties including fine and assembled collagen, collagen density, and collagen reticulation. Morphometric-FCS describes the collective morphometric traits of each individual fiber including fiber length, width, area, perimeter, and area to perimeter ratio. The morphometric score can further be segregated into fine and assembled fibers. Architecture-FCS includes entropy, inertia, correlation, and homogeneity to describe collagen fiber disorganization, compactness, patterns presence, distribution uniformity, and distortion of fibers.

The algorithm for the quantification of fibrosis in the clinical samples was adapted to MASH and LEAN hLiMTs. Only parameters and traits which were significantly changed in the MASH hLiMTs compared to the healthy LEAN control were selected for further analysis. Each collagen fiber was evaluated for histological traits such as fiber length, number of branches, and homogeneity, all of which were quantified. Additionally, collagen fibers were classified as “fine” or “assembled” based on the complexity of the collagen network, and the morphometric phenotypes which can be quantified for each subgroup. Assembled collagens appear as more reticulated collagen fiber network containing longer and thicker fibrils with many branches and nodes, whereas fine collagens are small and narrow collagen fibrils with minimal branches^24^. There are 32 histological phenotypic traits: collagen content (12 traits), collagen fiber morphometry (13 traits) and fibrosis architecture (7 traits). The histological traits were then evaluated further to determine a variety of statistical features, such as the mean, median and standard deviation. These are output as continuous variables defined as qFTs accounting for severity, progression, distortion, and variance for both fine and assembled collagens. A total of 316 qFTs were measured in MASH hLiMTs using FibroNest™ II platform, but only 43 qFTs were selected to generate the Ph-FCS. These 43 qFTs were preselected based on a calibration study that showed a significant changed in MASH hLiMTs vs LEAN or reference anti-fibrotic compound treatment. The 43 principal qFTs were distributed in collagen content (9), morphometric (18) and architectural (16) sub-phenotype parameters used for the assessment of severity of the disease (Supplementary Tables 1 and 2). The relative changes of each qFT between the microtissues and groups were visualized in the form of a heatmap. FibroNest™ analysis was conducted blindly to the SR-stained histological slides. The quantification of fibrosis upon treatment with ALK5i was conducted in three independent experiments to validate the algorithm. The quantification of fibrosis with reference anti-fibrotic compounds such as ALK5i and anti-TGF-β Ab was performed using FibroNest™ II software, where 43 qFTs were used and 7 to 10 microtissues per treatment group were analyzed. The optimized and next generation high sensitivity FibroNest™ III software was used for the quantification of fibrosis of clinical compounds Firsocostat and Selonsertib. FibroNest™ III software was able to detect even finer fibers of collagens than the FibroNest™ II platform. The quantification of fibrosis in optimized MASH hLiMTs using the highly sensitive and improved FibroNest™ III software enabled an increase of the number of significantly changed in MASH LiMTs vs LEAN or compound treatment qFTs from 43 to 206, covering many parameters defining the fine and assembled collagen. Out of 336 qFTs measured by FibroNest™ III software, 206 qFTs were changed in MASH vs LEAN hLiMTs and distributed among the various sub-phenotypes such as collagen content (10), morphometry (160) and architectural parameters (36) (Supplementary Table 3). For the quantification of fibrosis of MASH hLiMTs treated with clinical compounds 11 to 20 microtissues were used.

##### Creation of composite scores

Principal qFTs were automatically detected and combined into a normalized Ph-FCS. The Ph-FCS is a continuous fibrosis severity score (Ph-FCS, 1 to 10). The Ph-FCS is an aggregate of 3 sub-composite scores: the collagen-FCS, the morphometric-FCS and the architecture-FCS. Furthermore, each qFT is described individually for relative severity from least to most (green to red, respectively) in phenotypic heatmaps. A Brown-Forsythe version of one-way ANOVA with Welch’s correction in combination with Dunnett’s T3 multiple comparisons test vs MASH was used to calculate significant changes.

### Ethical compliance information

InSphero is working in compliance with the Swiss Federal Act on Research involving Human Beings (810.30) and does not require itself to receive ethical approval in using the sourced human biological post-mortem samples in the development and production of its biological products, because those samples were received anonymized. All the methods were carried out in accordance with relevant guidelines and regulations.

### Additional information

The contents of MSigDB are protected by copyright © 2004-2020 Broad Institute, Inc., Massachusetts Institute of Technology, and Regents of the University of California, subject to the terms and conditions of the Creative Commons Attribution 4.0 International License. MsigDB gene sets derived from KEGG pathways are protected by copyright, © 1995-2017 Kanehisa Laboratories, all rights reserved. EMA implementation of 3Rs principles: https://www.ema.europa.eu/en/homepage. FDA Technology Modernization Act 2.0: https://www.fda.gov/about-fda/reports/modernization-action-2022.

### Supplementary Information

Supplementary Information 1.

Supplementary Table 1.

Supplementary Table 2.

Supplementary Table 3.

Supplementary Table 4.

Supplementary Table 5.

## Data Availability

The dataset used and analyzed during the current study are available from the corresponding author on reasonable request.
