# Supplementary material for: Digital pathology with artificial intelligence analysis provides insight to the efficacy of anti-fibrotic compounds in human 3D MASH model
Source: Sci Rep. 2024 Mar 11;14:5885. doi: 10.1038/s41598-024-55438-2 (PMC10928082; doi:10.1038/s41598-024-55438-2)
Supplement: Supplementary file 1 — Supplementary Information 1. [file 41598_2024_55438_MOESM1_ESM.docx]

**Supplementary information**

**Supplementary figure 1.** Firsocostat and Selonsertib mono and combinatorial treatment decrease the deposition of fibrillated collagens in MASH hLiMT. (A) SR-BF images and fine and assembled collagen fibers are shown. For quantification of fibrosis n=11-20 tissues were used. The scan images are taken with 40x magnification, scale bar: 100 µm. (B) The quantification of fibrosis of LEAN, MASH and MASH hLiMTs treated with FIRSO, SELON and FIRSO with SELON on day 10 by FibroNest^TM^ III platform is presented. Purple is fine collagen fibers and blue are assembled and highly reticulated collagen fibers. The architecture is presented in accordance of the concentration of collagen (normalized collagen optical density), ranging from blue: faint collagen to red: dense collagen. Scale: 100µm

**Supplementary table 1.** ALK5i treatment decreases the deposition of fibrillated collagens in MASH hLiMT. The heatmap presents the 43 qFTs of FibroNest^TM^ II platform used for quantification of fibrosis upon treatment of MASH hLiMT with 0.5 µM ALK5i. In the heatmap, each row is a different qFTs composing the collagen, morphometric and architecture sub-phenotypes, whereas every column presents a different hLiMT (n=7-9) or regions of interest (ROIs) used for fibrosis quantification.

**Supplementary table 2.** Anti-TGF-ß Ab treatment decreases the deposition of fibrillated collagens in MASH hLiMTs. The heatmap presents the 43 qFTs of FibroNest^TM^ II platform used for quantification of fibrosis upon treatment of MASH hLiMT with 0.001 µM or 0.1 µM anti-TGF-ß Ab. In the heatmap, each row is a different qFTs composing the collagen, morphometric and architecture sub-phenotypes, whereas every column presents a different hLiMT (n=7-10) or ROIs used for fibrosis quantification.

**Supplementary table 3.** Firsocostat and Selonsertib mono and combinatorial treatment decrease the deposition of the fibrillated collagens. The heatmap presents the 206 qFTs of FibroNest^TM^ III platform used for quantification of fibrosis upon treatment of MASH hLiMT with FIRSO and SELON or their combination. In the heatmap every row presents a different qFT distributed in collagen, morphometric and architecture sub-phenotypes, whereas every column presents a different hLiMT (n=11-20) or ROIs used for fibrosis quantification.

**Supplementary table 4.** List of selected fibrosis related genes in MASH hLiMT over LEAN or compound treatment. The table specifies the official gene symbol, gene name and HUGO Gene Nomenclature Committee (HGNC) identifier. DEA results for these genes were extracted and presented in Figures 2, 4 and 7.

**Supplementary table 5**. List of selected fibrosis related gene sets in MASH hLiMT over LEAN or compound treatment. The table specifies the official gene set name, source, and members. GSEA results for these gene sets were extracted and presented in Figures 2, 4 and 7.


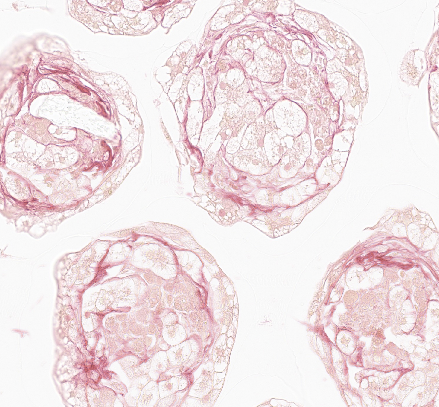

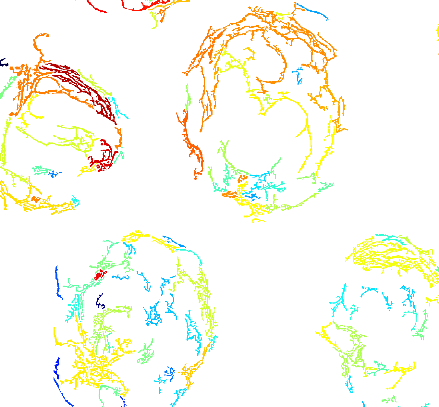

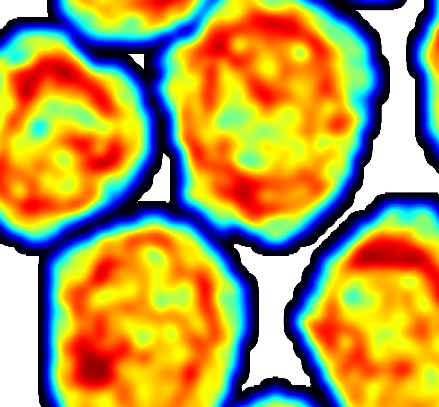

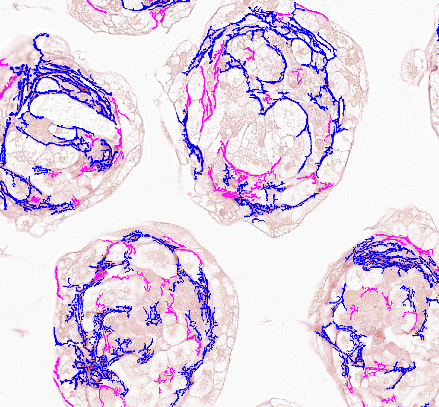

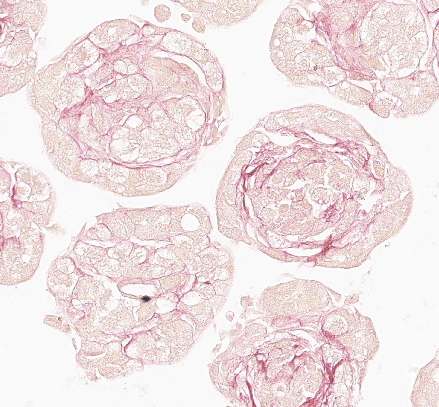

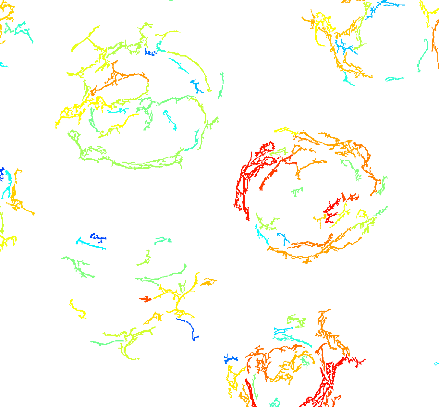

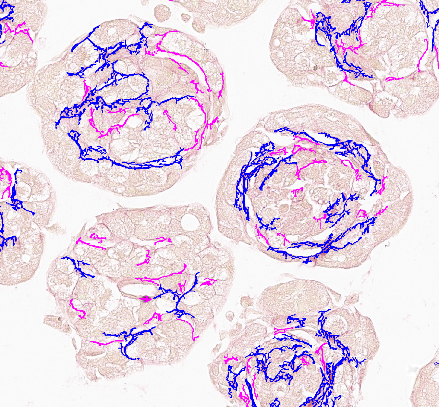

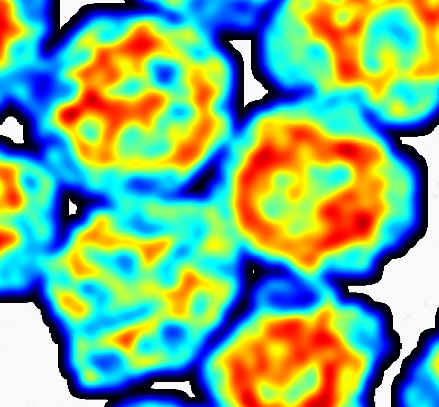

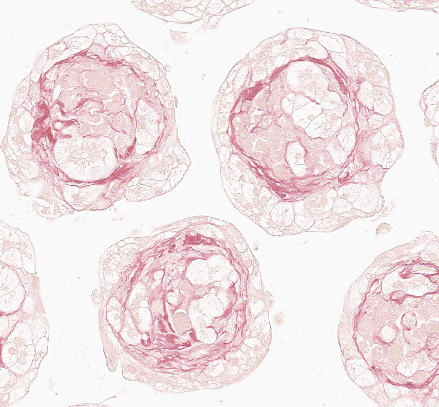

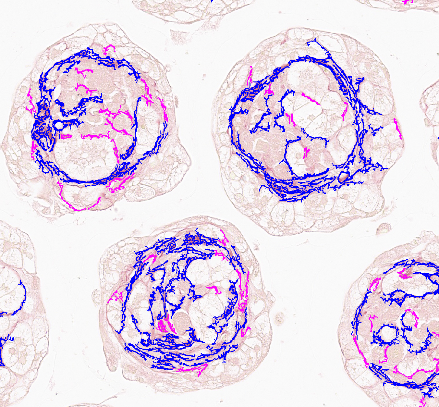

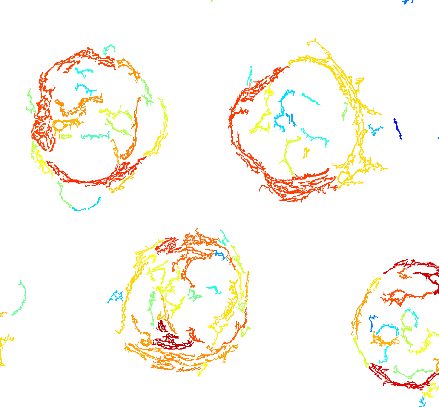

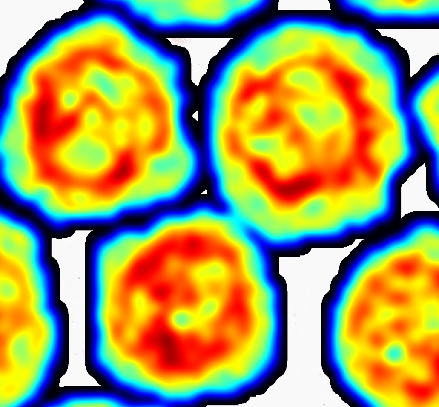

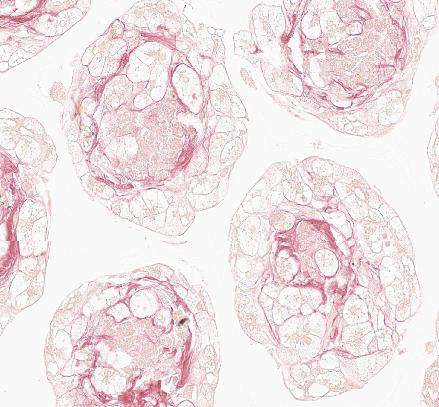

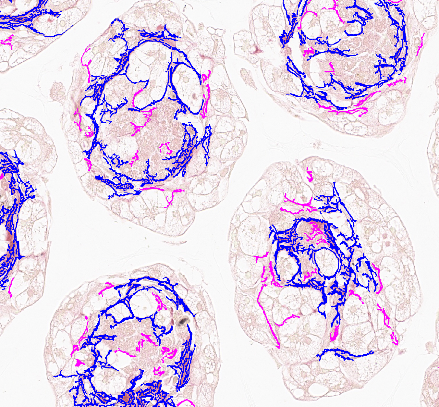

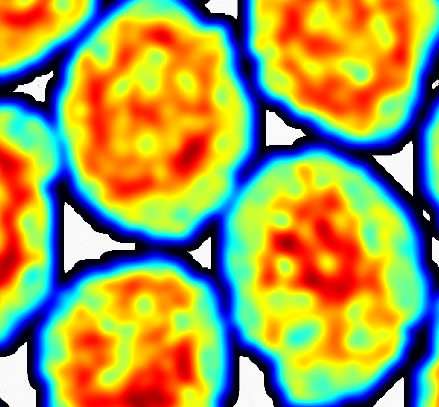

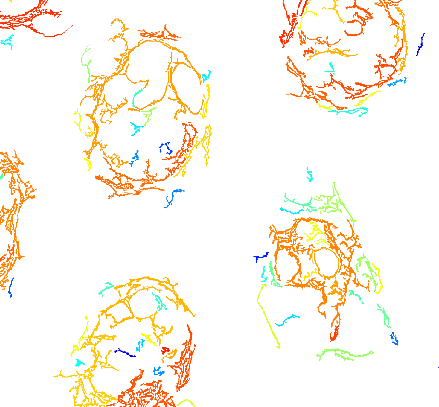

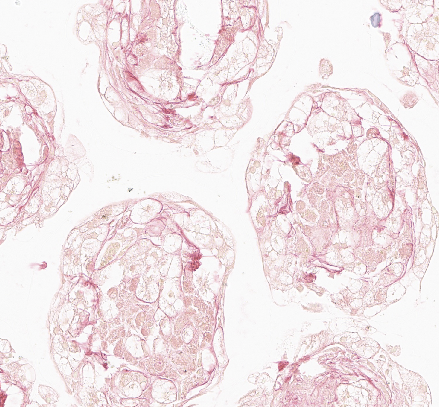

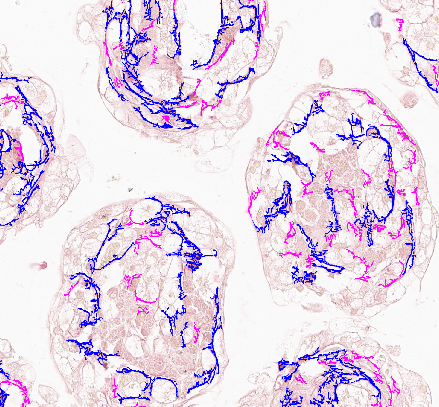

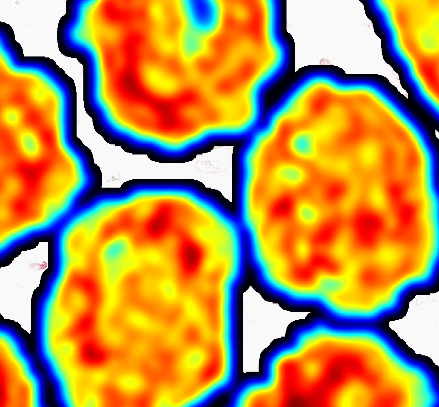

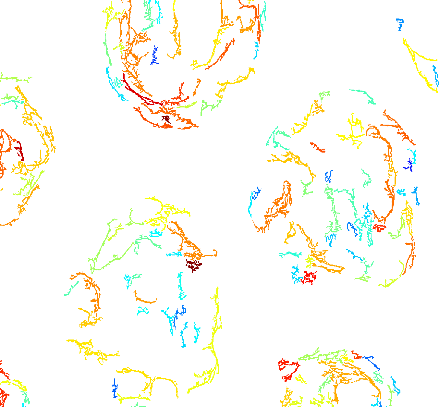

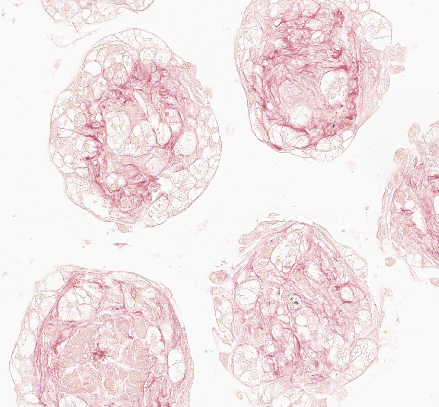

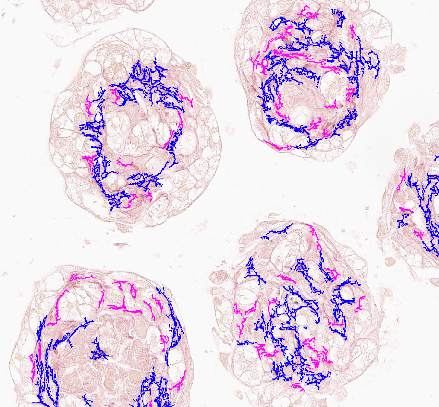

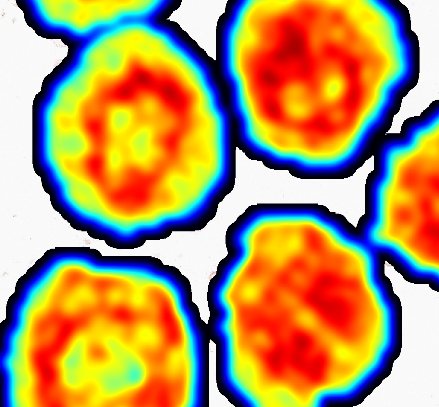

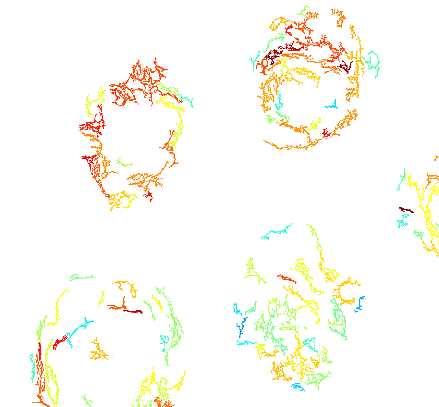

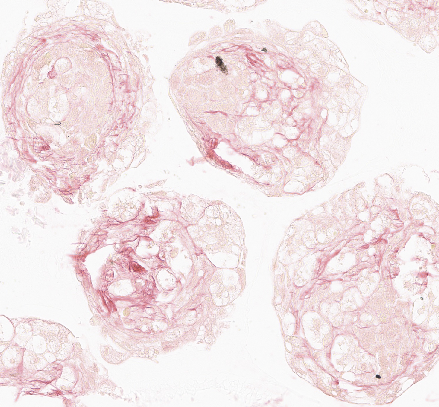

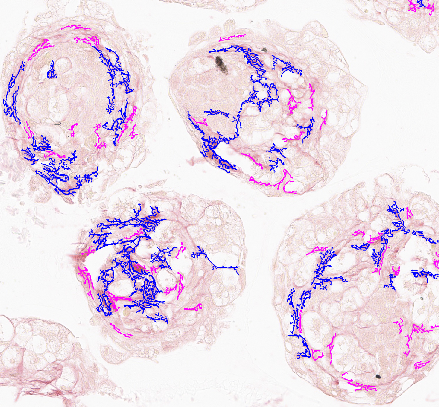

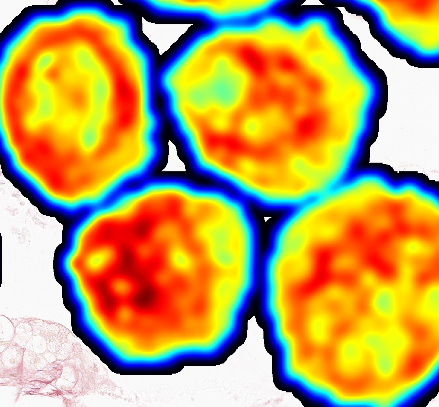

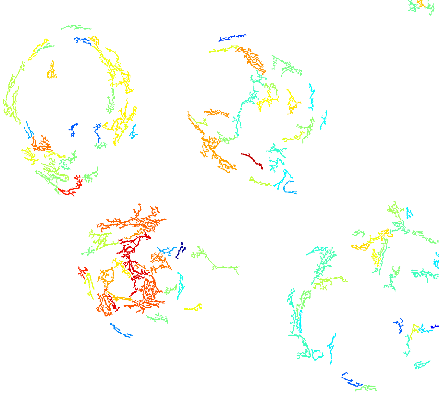


**Supplementary figure 1**

**Architecture**

**Density**

**SR-BF**

B

**Fine**

**Assembled**

**Collagen**

**LEAN**

**MASH**

**FIRSO [0.5µM]**

**FIRSO [10µM]**

**SELON [2 µM]**

**SELON [10µM]**

**FIRSO [0.5µM] SELON [10µM]**

A


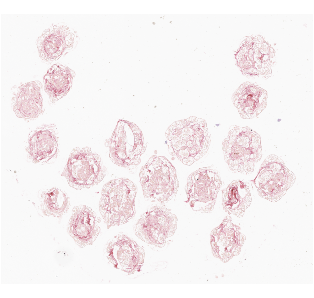

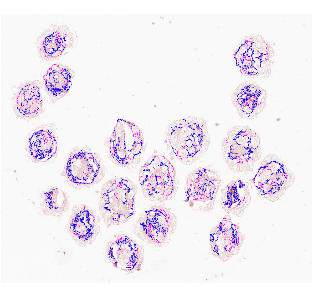

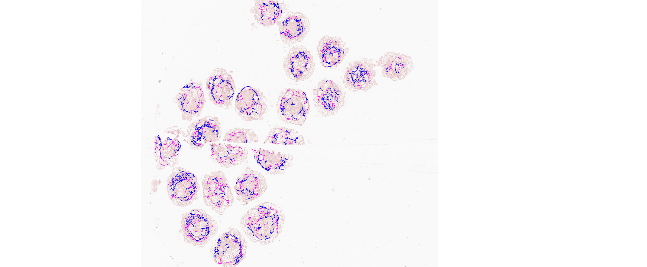

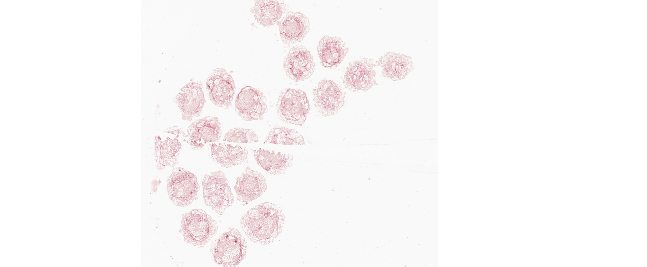

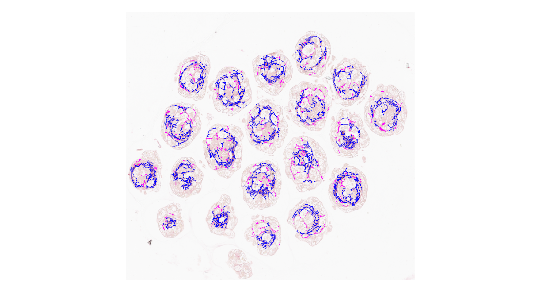

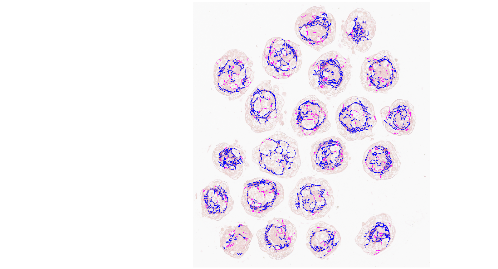

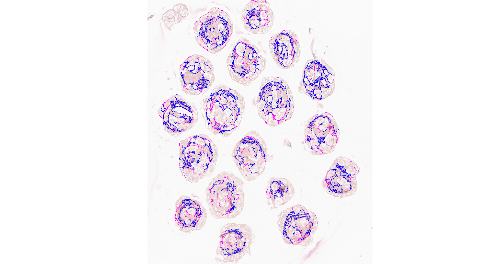

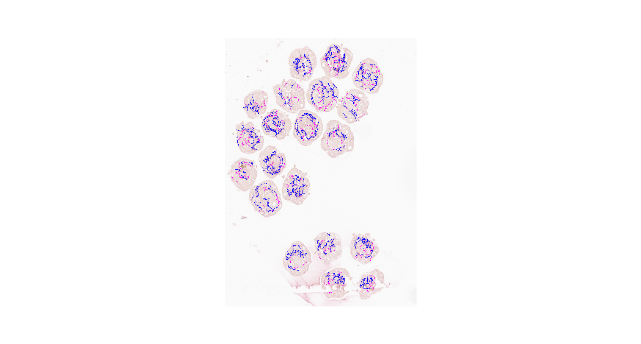

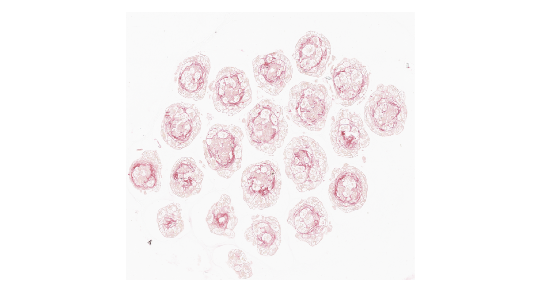

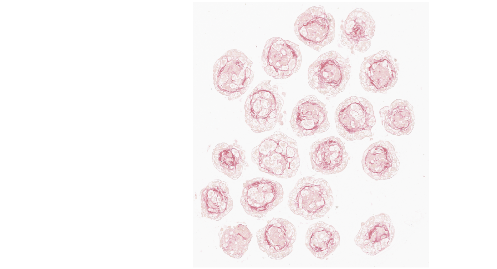

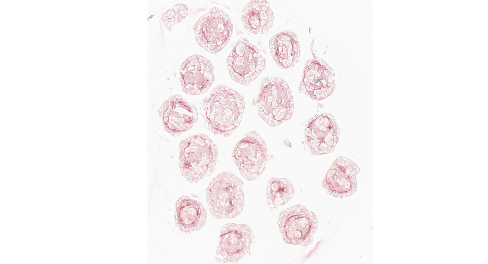

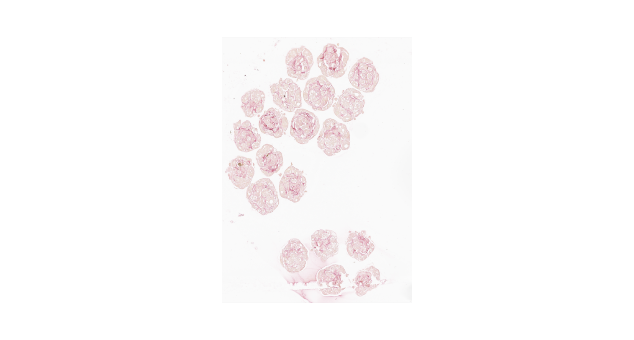

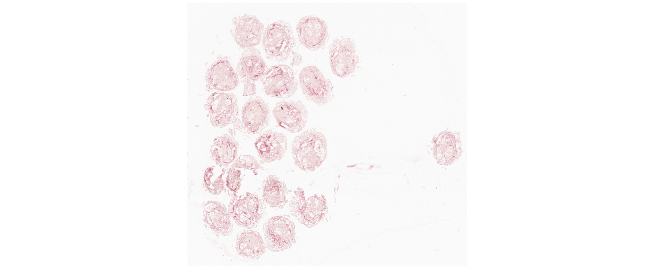

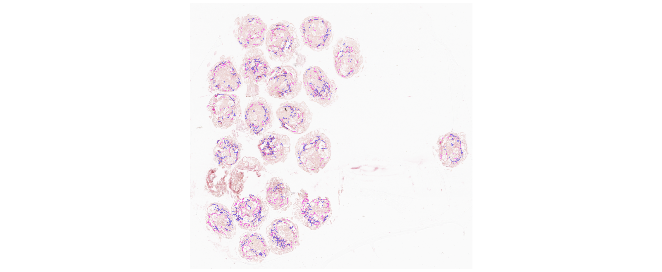


**Fine**

**Assembled**

**Collagen**

**SR-BF**

**LEAN**

**MASH**

**FIRSO [0.5µM]**

**FIRSO [10µM]**

**SELON [2 µM]**

**SELON [10µM]**

**FIRSO [0.5µM] SELON [10µM]**
